# Supplementary material for: Genome-wide identification, characterization and gene expression of BES1 transcription factor family in grapevine (Vitis vinifera L.)
Source: Sci Rep. 2023 Jan 5;13:240. doi: 10.1038/s41598-022-24407-y (PMC9816167; doi:10.1038/s41598-022-24407-y)
Supplement: Supplementary file 3 — Supplementary Information. [file 41598_2022_24407_MOESM3_ESM.zip › Vvi_Vvi/Vitis_vinifera.PN40024.v4.dna_sm.toplevel.fa.vs.Vitis_vinifera.PN40024.v4.dna_sm.toplevel.fa.html/Un.html]

|  |  |  |  |  |  |  |  |  |  |  |  |  |  |
| --- | --- | --- | --- | --- | --- | --- | --- | --- | --- | --- | --- | --- | --- |
| Duplication depth | Reference chromosome | Collinear blocks | | | | | | | | | | | |
| 0 | Vitvi00g04001\_t001 |  |  |  |  |  |  |  |  |  |  |  |  |
| 0 | Vitvi00g04002\_t001 |  |  |  |  |  |  |  |  |  |  |  |  |
| 0 | Vitvi00g04003\_t001 |  |  |  |  |  |  |  |  |  |  |  |  |
| 0 | Vitvi00g04005\_t001 |  |  |  |  |  |  |  |  |  |  |  |  |
| 0 | Vitvi00g04006\_t001 |  |  |  |  |  |  |  |  |  |  |  |  |
| 0 | Vitvi07g02863\_t002 |  |  |  |  |  |  |  |  |  |  |  |  |
| 0 | Vitvi07g02862\_t001 |  |  |  |  |  |  |  |  |  |  |  |  |
| 0 | Vitvi00g04007\_t003 |  |  |  |  |  |  |  |  |  |  |  |  |
| 0 | Vitvi07g02858\_t001 |  |  |  |  |  |  |  |  |  |  |  |  |
| 0 | Vitvi07g02853\_t001 |  |  |  |  |  |  |  |  |  |  |  |  |
| 0 | Vitvi07g02852\_t001 |  |  |  |  |  |  |  |  |  |  |  |  |
| 0 | Vitvi00g04008\_t001 |  |  |  |  |  |  |  |  |  |  |  |  |
| 0 | Vitvi00g04009\_t001 |  |  |  |  |  |  |  |  |  |  |  |  |
| 0 | Vitvi00g04010\_t001 |  |  |  |  |  |  |  |  |  |  |  |  |
| 0 | Vitvi00g04011\_t001 |  |  |  |  |  |  |  |  |  |  |  |  |
| 0 | Vitvi07g02842\_t001 |  |  |  |  |  |  |  |  |  |  |  |  |
| 0 | Vitvi07g03194\_t001 |  |  |  |  |  |  |  |  |  |  |  |  |
| 0 | Vitvi00g04012\_t001 |  |  |  |  |  |  |  |  |  |  |  |  |
| 0 | Vitvi07g03192\_t001 |  |  |  |  |  |  |  |  |  |  |  |  |
| 0 | Vitvi00g04013\_t001 |  |  |  |  |  |  |  |  |  |  |  |  |
| 0 | Vitvi00g04014\_t001 |  |  |  |  |  |  |  |  |  |  |  |  |
| 0 | Vitvi07g02918\_t001 |  |  |  |  |  |  |  |  |  |  |  |  |
| 0 | Vitvi00g04015\_t001 |  |  |  |  |  |  |  |  |  |  |  |  |
| 0 | Vitvi00g04016\_t001 |  |  |  |  |  |  |  |  |  |  |  |  |
| 0 | Vitvi00g04017\_t001 |  |  |  |  |  |  |  |  |  |  |  |  |
| 0 | Vitvi00g04018\_t001 |  |  |  |  |  |  |  |  |  |  |  |  |
| 0 | Vitvi07g02920\_t001 |  |  |  |  |  |  |  |  |  |  |  |  |
| 0 | Vitvi00g04019\_t001 |  |  |  |  |  |  |  |  |  |  |  |  |
| 0 | Vitvi00g04020\_t001 |  |  |  |  |  |  |  |  |  |  |  |  |
| 0 | Vitvi00g04021\_t001 |  |  |  |  |  |  |  |  |  |  |  |  |
| 0 | Vitvi00g04022\_t001 |  |  |  |  |  |  |  |  |  |  |  |  |
| 0 | Vitvi00g04023\_t001 |  |  |  |  |  |  |  |  |  |  |  |  |
| 0 | Vitvi00g04024\_t001 |  |  |  |  |  |  |  |  |  |  |  |  |
| 0 | Vitvi00g04025\_t001 |  |  |  |  |  |  |  |  |  |  |  |  |
| 0 | Vitvi07g02923\_t001 |  |  |  |  |  |  |  |  |  |  |  |  |
| 0 | Vitvi00g04026\_t001 |  |  |  |  |  |  |  |  |  |  |  |  |
| 0 | Vitvi00g04027\_t001 |  |  |  |  |  |  |  |  |  |  |  |  |
| 0 | Vitvi00g04028\_t001 |  |  |  |  |  |  |  |  |  |  |  |  |
| 0 | Vitvi00g04029\_t001 |  |  |  |  |  |  |  |  |  |  |  |  |
| 0 | Vitvi00g04030\_t001 |  |  |  |  |  |  |  |  |  |  |  |  |
| 0 | Vitvi00g04031\_t001 |  |  |  |  |  |  |  |  |  |  |  |  |
| 0 | Vitvi00g04032\_t001 |  |  |  |  |  |  |  |  |  |  |  |  |
| 0 | Vitvi00g04033\_t001 |  |  |  |  |  |  |  |  |  |  |  |  |
| 0 | Vitvi00g04034\_t001 |  |  |  |  |  |  |  |  |  |  |  |  |
| 0 | Vitvi00g04035\_t001 |  |  |  |  |  |  |  |  |  |  |  |  |
| 0 | Vitvi00g04036\_t001 |  |  |  |  |  |  |  |  |  |  |  |  |
| 0 | Vitvi00g04037\_t001 |  |  |  |  |  |  |  |  |  |  |  |  |
| 0 | Vitvi02g01769\_t001 |  |  |  |  |  |  |  |  |  |  |  |  |
| 0 | Vitvi02g01770\_t004 |  |  |  |  |  |  |  |  |  |  |  |  |
| 0 | Vitvi02g01771\_t001 |  |  |  |  |  |  |  |  |  |  |  |  |
| 0 | Vitvi02g01773\_t001 |  |  |  |  |  |  |  |  |  |  |  |  |
| 0 | Vitvi02g01774\_t001 |  |  |  |  |  |  |  |  |  |  |  |  |
| 0 | Vitvi02g01775\_t001 |  |  |  |  |  |  |  |  |  |  |  |  |
| 0 | Vitvi02g01776\_t001 |  |  |  |  |  |  |  |  |  |  |  |  |
| 0 | Vitvi02g01777\_t001 |  |  |  |  |  |  |  |  |  |  |  |  |
| 0 | Vitvi02g01778\_t001 |  |  |  |  |  |  |  |  |  |  |  |  |
| 0 | Vitvi02g01779\_t001 |  |  |  |  |  |  |  |  |  |  |  |  |
| 0 | Vitvi02g01780\_t001 |  |  |  |  |  |  |  |  |  |  |  |  |
| 0 | Vitvi00g04038\_t001 |  |  |  |  |  |  |  |  |  |  |  |  |
| 0 | Vitvi00g04039\_t001 |  |  |  |  |  |  |  |  |  |  |  |  |
| 0 | Vitvi00g04040\_t001 |  |  |  |  |  |  |  |  |  |  |  |  |
| 0 | Vitvi00g04041\_t002 |  |  |  |  |  |  |  |  |  |  |  |  |
| 0 | Vitvi00g04042\_t004 |  |  |  |  |  |  |  |  |  |  |  |  |
| 0 | Vitvi00g04043\_t001 |  |  |  |  |  |  |  |  |  |  |  |  |
| 0 | Vitvi00g04044\_t001 |  |  |  |  |  |  |  |  |  |  |  |  |
| 0 | Vitvi00g04045\_t001 |  |  |  |  |  |  |  |  |  |  |  |  |
| 0 | Vitvi02g01739\_t001 |  |  |  |  |  |  |  |  |  |  |  |  |
| 0 | Vitvi00g04046\_t001 |  |  |  |  |  |  |  |  |  |  |  |  |
| 0 | Vitvi02g01738\_t001 |  |  |  |  |  |  |  |  |  |  |  |  |
| 0 | Vitvi02g01736\_t001 |  |  |  |  |  |  |  |  |  |  |  |  |
| 0 | Vitvi02g01735\_t001 |  |  |  |  |  |  |  |  |  |  |  |  |
| 0 | Vitvi00g04047\_t001 |  |  |  |  |  |  |  |  |  |  |  |  |
| 0 | Vitvi02g01733\_t001 |  |  |  |  |  |  |  |  |  |  |  |  |
| 0 | Vitvi02g01732\_t001 |  |  |  |  |  |  |  |  |  |  |  |  |
| 0 | Vitvi00g04048\_t001 |  |  |  |  |  |  |  |  |  |  |  |  |
| 0 | Vitvi00g04049\_t001 |  |  |  |  |  |  |  |  |  |  |  |  |
| 0 | Vitvi02g01729\_t001 |  |  |  |  |  |  |  |  |  |  |  |  |
| 0 | Vitvi00g04050\_t001 |  |  |  |  |  |  |  |  |  |  |  |  |
| 0 | Vitvi00g04051\_t001 |  |  |  |  |  |  |  |  |  |  |  |  |
| 0 | Vitvi00g04052\_t001 |  |  |  |  |  |  |  |  |  |  |  |  |
| 2 | Vitvi00g04053\_t001 |  | Vitvi09g04424\_t001 |  | Vitvi09g04408\_t001 |  |  |  |  |  |  |  |  |
| 2 | Vitvi00g04054\_t001 |  | Vitvi09g01823\_t001 |  | \| \| |  |  |  |  |  |  |  |  |
| 2 | Vitvi00g04055\_t001 |  | \| \| |  | \| \| |  |  |  |  |  |  |  |  |
| 2 | Vitvi00g04056\_t001 |  | \| \| |  | \| \| |  |  |  |  |  |  |  |  |
| 2 | Vitvi00g04057\_t001 |  | \| \| |  | Vitvi09g04412\_t001 |  |  |  |  |  |  |  |  |
| 2 | Vitvi00g04058\_t001 |  | \| \| |  | Vitvi09g04413\_t001 |  |  |  |  |  |  |  |  |
| 2 | Vitvi00g04059\_t001 |  | Vitvi09g04422\_t001 |  | \| \| |  |  |  |  |  |  |  |  |
| 2 | Vitvi00g04060\_t001 |  | \| \| |  | \| \| |  |  |  |  |  |  |  |  |
| 3 | Vitvi00g04061\_t001 |  | \| \| |  | \| \| |  | Vitvi00g04152\_t001 |  |  |  |  |  |  |
| 3 | Vitvi00g04062\_t001 |  | \| \| |  | \| \| |  | \| \| |  |  |  |  |  |  |
| 3 | Vitvi00g04063\_t001 |  | Vitvi09g01827\_t001 |  | Vitvi09g01827\_t001 |  | \| \| |  |  |  |  |  |  |
| 3 | Vitvi00g04064\_t001 |  | \| \| |  | \| \| |  | \| \| |  |  |  |  |  |  |
| 3 | Vitvi00g04065\_t001 |  | \| \| |  | \| \| |  | \| \| |  |  |  |  |  |  |
| 3 | Vitvi00g04066\_t001 |  | \| \| |  | \| \| |  | \| \| |  |  |  |  |  |  |
| 3 | Vitvi00g04067\_t001 |  | Vitvi09g04416\_t001 |  | Vitvi09g04421\_t001 |  | \| \| |  |  |  |  |  |  |
| 3 | Vitvi00g04068\_t001 |  | \| \| |  | Vitvi09g04422\_t001 |  | \| \| |  |  |  |  |  |  |
| 2 | Vitvi00g04069\_t001 |  | Vitvi09g04415\_t001 |  |  |  | \| \| |  |  |  |  |  |  |
| 1 | Vitvi00g04070\_t001 |  |  |  |  |  | \| \| |  |  |  |  |  |  |
| 1 | Vitvi00g04071\_t001 |  |  |  |  |  | \| \| |  |  |  |  |  |  |
| 1 | Vitvi00g04072\_t001 |  |  |  |  |  | \| \| |  |  |  |  |  |  |
| 1 | Vitvi00g04073\_t001 |  |  |  |  |  | \| \| |  |  |  |  |  |  |
| 1 | Vitvi00g04074\_t001 |  |  |  |  |  | \| \| |  |  |  |  |  |  |
| 1 | Vitvi00g04075\_t001 |  |  |  |  |  | \| \| |  |  |  |  |  |  |
| 1 | Vitvi00g04076\_t001 |  |  |  |  |  | \| \| |  |  |  |  |  |  |
| 1 | Vitvi00g04077\_t001 |  |  |  |  |  | \| \| |  |  |  |  |  |  |
| 1 | Vitvi00g04078\_t001 |  |  |  |  |  | \| \| |  |  |  |  |  |  |
| 1 | Vitvi00g04079\_t001 |  |  |  |  |  | \| \| |  |  |  |  |  |  |
| 1 | Vitvi00g04080\_t001 |  |  |  |  |  | \| \| |  |  |  |  |  |  |
| 1 | Vitvi00g04081\_t001 |  |  |  |  |  | \| \| |  |  |  |  |  |  |
| 1 | Vitvi00g04082\_t001 |  |  |  |  |  | \| \| |  |  |  |  |  |  |
| 1 | Vitvi00g04083\_t001 |  |  |  |  |  | \| \| |  |  |  |  |  |  |
| 2 | Vitvi00g04084\_t001 |  | Vitvi03g04346\_t001 |  |  |  | Vitvi00g04156\_t001 |  |  |  |  |  |  |
| 2 | Vitvi00g04085\_t001 |  | \| \| |  |  |  | \| \| |  |  |  |  |  |  |
| 2 | Vitvi00g04086\_t001 |  | \| \| |  |  |  | \| \| |  |  |  |  |  |  |
| 2 | Vitvi00g04087\_t001 |  | \| \| |  |  |  | \| \| |  |  |  |  |  |  |
| 2 | Vitvi00g04088\_t001 |  | \| \| |  |  |  | \| \| |  |  |  |  |  |  |
| 2 | Vitvi00g04089\_t001 |  | \| \| |  |  |  | \| \| |  |  |  |  |  |  |
| 2 | Vitvi00g04090\_t001 |  | \| \| |  |  |  | \| \| |  |  |  |  |  |  |
| 2 | Vitvi00g04091\_t001 |  | \| \| |  |  |  | \| \| |  |  |  |  |  |  |
| 2 | Vitvi00g04092\_t001 |  | \| \| |  |  |  | \| \| |  |  |  |  |  |  |
| 2 | Vitvi00g04093\_t001 |  | \| \| |  |  |  | \| \| |  |  |  |  |  |  |
| 2 | Vitvi00g04094\_t001 |  | \| \| |  |  |  | \| \| |  |  |  |  |  |  |
| 2 | Vitvi00g04095\_t001 |  | \| \| |  |  |  | \| \| |  |  |  |  |  |  |
| 2 | Vitvi00g04096\_t001 |  | \| \| |  |  |  | \| \| |  |  |  |  |  |  |
| 2 | Vitvi00g04097\_t001 |  | \| \| |  |  |  | \| \| |  |  |  |  |  |  |
| 4 | Vitvi00g04098\_t001 |  | \| \| |  | Vitvi02g01542\_t001 |  | Vitvi00g04168\_t001 |  | Vitvi09g04397\_t001 |  |  |  |  |
| 4 | Vitvi00g04099\_t001 |  | \| \| |  | Vitvi02g00896\_t001 |  | Vitvi00g04169\_t001 |  | \| \| |  |  |  |  |
| 4 | Vitvi00g04100\_t001 |  | \| \| |  | \| \| |  | Vitvi00g04170\_t001 |  | Vitvi09g04412\_t001 |  |  |  |  |
| 4 | Vitvi00g04101\_t001 |  | \| \| |  | Vitvi02g00897\_t001 |  | Vitvi04g02271\_t001 |  | Vitvi09g04413\_t001 |  |  |  |  |
| 4 | Vitvi00g04102\_t001 |  | \| \| |  | \| \| |  | Vitvi04g02269\_t001 |  | \| \| |  |  |  |  |
| 3 | Vitvi00g04103\_t001 |  | \| \| |  | Vitvi02g04258\_t001 |  |  |  | \| \| |  |  |  |  |
| 3 | Vitvi00g04104\_t001 |  | \| \| |  | Vitvi02g04259\_t001 |  |  |  | \| \| |  |  |  |  |
| 3 | Vitvi00g04105\_t001 |  | \| \| |  | Vitvi02g04260\_t001 |  |  |  | Vitvi09g04417\_t001 |  |  |  |  |
| 3 | Vitvi00g04106\_t001 |  | \| \| |  | Vitvi02g04261\_t001 |  |  |  | Vitvi09g04418\_t001 |  |  |  |  |
| 3 | Vitvi00g04107\_t001 |  | \| \| |  | \| \| |  |  |  | Vitvi09g04419\_t001 |  |  |  |  |
| 2 | Vitvi00g04108\_t001 |  | Vitvi03g04333\_t001 |  | \| \| |  |  |  |  |  |  |  |  |
| 3 | Vitvi00g04109\_t001 |  | Vitvi03g04330\_t001 |  | \| \| |  | Vitvi03g04348\_t001 |  |  |  |  |  |  |
| 3 | Vitvi00g04110\_t001 |  | \| \| |  | \| \| |  | Vitvi03g04346\_t001 |  |  |  |  |  |  |
| 3 | Vitvi00g04111\_t001 |  | Vitvi03g04328\_t001 |  | \| \| |  | \| \| |  |  |  |  |  |  |
| 3 | Vitvi00g04112\_t001 |  | \| \| |  | \| \| |  | \| \| |  |  |  |  |  |  |
| 3 | Vitvi00g04113\_t001 |  | \| \| |  | \| \| |  | \| \| |  |  |  |  |  |  |
| 3 | Vitvi00g04114\_t001 |  | Vitvi03g04327\_t001 |  | \| \| |  | Vitvi03g04345\_t001 |  |  |  |  |  |  |
| 3 | Vitvi00g04115\_t001 |  | \| \| |  | \| \| |  | Vitvi03g04344\_t001 |  |  |  |  |  |  |
| 3 | Vitvi00g04116\_t001 |  | \| \| |  | \| \| |  | \| \| |  |  |  |  |  |  |
| 3 | Vitvi00g04117\_t001 |  | Vitvi03g01765\_t001 |  | \| \| |  | Vitvi03g04343\_t001 |  |  |  |  |  |  |
| 2 | Vitvi00g04118\_t001 |  |  |  | \| \| |  | Vitvi03g04342\_t001 |  |  |  |  |  |  |
| 2 | Vitvi00g04119\_t001 |  |  |  | \| \| |  | Vitvi03g04341\_t001 |  |  |  |  |  |  |
| 2 | Vitvi00g04120\_t001 |  |  |  | \| \| |  | Vitvi03g04340\_t001 |  |  |  |  |  |  |
| 2 | Vitvi00g04121\_t001 |  |  |  | \| \| |  | Vitvi03g04339\_t001 |  |  |  |  |  |  |
| 2 | Vitvi00g04122\_t001 |  |  |  | \| \| |  | Vitvi03g04335\_t001 |  |  |  |  |  |  |
| 2 | Vitvi00g04123\_t001 |  |  |  | \| \| |  | \| \| |  |  |  |  |  |  |
| 2 | Vitvi00g04124\_t001 |  |  |  | \| \| |  | \| \| |  |  |  |  |  |  |
| 2 | Vitvi00g04125\_t001 |  |  |  | \| \| |  | Vitvi03g04332\_t001 |  |  |  |  |  |  |
| 1 | Vitvi00g04126\_t001 |  |  |  | \| \| |  |  |  |  |  |  |  |  |
| 1 | Vitvi00g04127\_t001 |  |  |  | \| \| |  |  |  |  |  |  |  |  |
| 1 | Vitvi00g04128\_t001 |  |  |  | \| \| |  |  |  |  |  |  |  |  |
| 1 | Vitvi00g04129\_t001 |  |  |  | \| \| |  |  |  |  |  |  |  |  |
| 1 | Vitvi00g04130\_t001 |  |  |  | \| \| |  |  |  |  |  |  |  |  |
| 1 | Vitvi00g04131\_t001 |  |  |  | Vitvi02g04280\_t001 |  |  |  |  |  |  |  |  |
| 0 | Vitvi00g04132\_t001 |  |  |  |  |  |  |  |  |  |  |  |  |
| 0 | Vitvi00g04133\_t001 |  |  |  |  |  |  |  |  |  |  |  |  |
| 0 | Vitvi00g04134\_t001 |  |  |  |  |  |  |  |  |  |  |  |  |
| 0 | Vitvi00g04135\_t001 |  |  |  |  |  |  |  |  |  |  |  |  |
| 0 | Vitvi00g04136\_t001 |  |  |  |  |  |  |  |  |  |  |  |  |
| 0 | Vitvi00g04137\_t001 |  |  |  |  |  |  |  |  |  |  |  |  |
| 0 | Vitvi00g04138\_t001 |  |  |  |  |  |  |  |  |  |  |  |  |
| 0 | Vitvi00g04139\_t001 |  |  |  |  |  |  |  |  |  |  |  |  |
| 0 | Vitvi02g01784\_t001 |  |  |  |  |  |  |  |  |  |  |  |  |
| 0 | Vitvi02g01786\_t001 |  |  |  |  |  |  |  |  |  |  |  |  |
| 0 | Vitvi02g01788\_t001 |  |  |  |  |  |  |  |  |  |  |  |  |
| 0 | Vitvi02g01789\_t001 |  |  |  |  |  |  |  |  |  |  |  |  |
| 0 | Vitvi00g04140\_t001 |  |  |  |  |  |  |  |  |  |  |  |  |
| 0 | Vitvi00g04141\_t001 |  |  |  |  |  |  |  |  |  |  |  |  |
| 0 | Vitvi00g04142\_t001 |  |  |  |  |  |  |  |  |  |  |  |  |
| 0 | Vitvi02g01794\_t001 |  |  |  |  |  |  |  |  |  |  |  |  |
| 0 | Vitvi00g04143\_t001 |  |  |  |  |  |  |  |  |  |  |  |  |
| 0 | Vitvi00g04144\_t001 |  |  |  |  |  |  |  |  |  |  |  |  |
| 0 | Vitvi00g04145\_t002 |  |  |  |  |  |  |  |  |  |  |  |  |
| 0 | Vitvi00g04146\_t002 |  |  |  |  |  |  |  |  |  |  |  |  |
| 0 | Vitvi02g01800\_t001 |  |  |  |  |  |  |  |  |  |  |  |  |
| 0 | Vitvi00g04147\_t001 |  |  |  |  |  |  |  |  |  |  |  |  |
| 0 | Vitvi02g01805\_t001 |  |  |  |  |  |  |  |  |  |  |  |  |
| 0 | Vitvi02g01806\_t001 |  |  |  |  |  |  |  |  |  |  |  |  |
| 0 | Vitvi02g01809\_t001 |  |  |  |  |  |  |  |  |  |  |  |  |
| 0 | Vitvi02g01810\_t001 |  |  |  |  |  |  |  |  |  |  |  |  |
| 0 | Vitvi02g01811\_t001 |  |  |  |  |  |  |  |  |  |  |  |  |
| 0 | Vitvi02g01812\_t001 |  |  |  |  |  |  |  |  |  |  |  |  |
| 0 | Vitvi00g04148\_t001 |  |  |  |  |  |  |  |  |  |  |  |  |
| 0 | Vitvi00g04149\_t001 |  |  |  |  |  |  |  |  |  |  |  |  |
| 0 | Vitvi00g04150\_t001 |  |  |  |  |  |  |  |  |  |  |  |  |
| 0 | Vitvi00g04151\_t001 |  |  |  |  |  |  |  |  |  |  |  |  |
| 1 | Vitvi00g04152\_t001 |  | Vitvi00g04061\_t001 |  |  |  |  |  |  |  |  |  |  |
| 1 | Vitvi00g04153\_t001 |  | \| \| |  |  |  |  |  |  |  |  |  |  |
| 1 | Vitvi00g04154\_t001 |  | \| \| |  |  |  |  |  |  |  |  |  |  |
| 1 | Vitvi00g04155\_t001 |  | \| \| |  |  |  |  |  |  |  |  |  |  |
| 1 | Vitvi00g04156\_t001 |  | Vitvi00g04084\_t001 |  |  |  |  |  |  |  |  |  |  |
| 1 | Vitvi00g04157\_t001 |  | \| \| |  |  |  |  |  |  |  |  |  |  |
| 1 | Vitvi00g04158\_t001 |  | \| \| |  |  |  |  |  |  |  |  |  |  |
| 1 | Vitvi00g04159\_t001 |  | \| \| |  |  |  |  |  |  |  |  |  |  |
| 1 | Vitvi00g04160\_t001 |  | \| \| |  |  |  |  |  |  |  |  |  |  |
| 1 | Vitvi00g04161\_t001 |  | \| \| |  |  |  |  |  |  |  |  |  |  |
| 1 | Vitvi00g04162\_t001 |  | \| \| |  |  |  |  |  |  |  |  |  |  |
| 1 | Vitvi00g04163\_t001 |  | \| \| |  |  |  |  |  |  |  |  |  |  |
| 1 | Vitvi00g04164\_t001 |  | \| \| |  |  |  |  |  |  |  |  |  |  |
| 1 | Vitvi00g04165\_t001 |  | \| \| |  |  |  |  |  |  |  |  |  |  |
| 1 | Vitvi00g04166\_t001 |  | \| \| |  |  |  |  |  |  |  |  |  |  |
| 1 | Vitvi00g04167\_t001 |  | \| \| |  |  |  |  |  |  |  |  |  |  |
| 1 | Vitvi00g04168\_t001 |  | Vitvi00g04098\_t001 |  |  |  |  |  |  |  |  |  |  |
| 1 | Vitvi00g04169\_t001 |  | Vitvi00g04099\_t001 |  |  |  |  |  |  |  |  |  |  |
| 1 | Vitvi00g04170\_t001 |  | Vitvi00g04100\_t001 |  |  |  |  |  |  |  |  |  |  |
| 1 | Vitvi04g02271\_t001 |  | Vitvi00g04101\_t001 |  |  |  |  |  |  |  |  |  |  |
| 1 | Vitvi04g02269\_t001 |  | Vitvi00g04102\_t001 |  |  |  |  |  |  |  |  |  |  |
| 0 | Vitvi04g02263\_t001 |  |  |  |  |  |  |  |  |  |  |  |  |
| 0 | Vitvi00g04171\_t001 |  |  |  |  |  |  |  |  |  |  |  |  |
| 0 | Vitvi00g04172\_t001 |  |  |  |  |  |  |  |  |  |  |  |  |
| 0 | Vitvi00g04173\_t001 |  |  |  |  |  |  |  |  |  |  |  |  |
| 0 | Vitvi07g02901\_t001 |  |  |  |  |  |  |  |  |  |  |  |  |
| 0 | Vitvi07g02900\_t001 |  |  |  |  |  |  |  |  |  |  |  |  |
| 0 | Vitvi00g04174\_t001 |  |  |  |  |  |  |  |  |  |  |  |  |
| 0 | Vitvi07g02897\_t001 |  |  |  |  |  |  |  |  |  |  |  |  |
| 0 | Vitvi07g02896\_t001 |  |  |  |  |  |  |  |  |  |  |  |  |
| 0 | Vitvi07g02895\_t001 |  |  |  |  |  |  |  |  |  |  |  |  |
| 0 | Vitvi07g02894\_t001 |  |  |  |  |  |  |  |  |  |  |  |  |
| 0 | Vitvi00g04175\_t001 |  |  |  |  |  |  |  |  |  |  |  |  |
| 0 | Vitvi07g02893\_t001 |  |  |  |  |  |  |  |  |  |  |  |  |
| 0 | Vitvi00g04176\_t001 |  |  |  |  |  |  |  |  |  |  |  |  |
| 0 | Vitvi00g04177\_t001 |  |  |  |  |  |  |  |  |  |  |  |  |
| 0 | Vitvi00g04178\_t001 |  |  |  |  |  |  |  |  |  |  |  |  |
| 0 | Vitvi00g04179\_t001 |  |  |  |  |  |  |  |  |  |  |  |  |
| 0 | Vitvi00g04180\_t001 |  |  |  |  |  |  |  |  |  |  |  |  |
| 0 | Vitvi00g04181\_t001 |  |  |  |  |  |  |  |  |  |  |  |  |
| 0 | Vitvi01g02305\_t001 |  |  |  |  |  |  |  |  |  |  |  |  |
| 0 | Vitvi00g04182\_t001 |  |  |  |  |  |  |  |  |  |  |  |  |
| 0 | Vitvi00g04183\_t001 |  |  |  |  |  |  |  |  |  |  |  |  |
| 0 | Vitvi00g04184\_t001 |  |  |  |  |  |  |  |  |  |  |  |  |
| 0 | Vitvi00g04185\_t001 |  |  |  |  |  |  |  |  |  |  |  |  |
| 0 | Vitvi00g04186\_t001 |  |  |  |  |  |  |  |  |  |  |  |  |
| 0 | Vitvi00g04187\_t001 |  |  |  |  |  |  |  |  |  |  |  |  |
| 0 | Vitvi00g04188\_t001 |  |  |  |  |  |  |  |  |  |  |  |  |
| 0 | Vitvi00g04189\_t001 |  |  |  |  |  |  |  |  |  |  |  |  |
| 0 | Vitvi00g04190\_t001 |  |  |  |  |  |  |  |  |  |  |  |  |
| 0 | Vitvi00g04191\_t001 |  |  |  |  |  |  |  |  |  |  |  |  |
| 0 | Vitvi01g02307\_t001 |  |  |  |  |  |  |  |  |  |  |  |  |
| 0 | Vitvi00g04192\_t001 |  |  |  |  |  |  |  |  |  |  |  |  |
| 0 | Vitvi00g04193\_t001 |  |  |  |  |  |  |  |  |  |  |  |  |
| 0 | Vitvi00g04194\_t001 |  |  |  |  |  |  |  |  |  |  |  |  |
| 0 | Vitvi00g04195\_t001 |  |  |  |  |  |  |  |  |  |  |  |  |
| 0 | Vitvi00g04196\_t001 |  |  |  |  |  |  |  |  |  |  |  |  |
| 0 | Vitvi00g04197\_t001 |  |  |  |  |  |  |  |  |  |  |  |  |
| 0 | Vitvi00g04198\_t001 |  |  |  |  |  |  |  |  |  |  |  |  |
| 0 | Vitvi00g04199\_t001 |  |  |  |  |  |  |  |  |  |  |  |  |
| 0 | Vitvi00g04200\_t001 |  |  |  |  |  |  |  |  |  |  |  |  |
| 0 | Vitvi00g04201\_t001 |  |  |  |  |  |  |  |  |  |  |  |  |
| 0 | Vitvi00g04202\_t001 |  |  |  |  |  |  |  |  |  |  |  |  |
| 0 | Vitvi00g04203\_t001 |  |  |  |  |  |  |  |  |  |  |  |  |
| 0 | Vitvi00g04204\_t001 |  |  |  |  |  |  |  |  |  |  |  |  |
| 0 | Vitvi00g04205\_t001 |  |  |  |  |  |  |  |  |  |  |  |  |
| 0 | Vitvi00g04206\_t001 |  |  |  |  |  |  |  |  |  |  |  |  |
| 0 | Vitvi09g02088\_t001 |  |  |  |  |  |  |  |  |  |  |  |  |
| 0 | Vitvi00g04207\_t001 |  |  |  |  |  |  |  |  |  |  |  |  |
| 0 | Vitvi00g04208\_t001 |  |  |  |  |  |  |  |  |  |  |  |  |
| 0 | Vitvi00g04209\_t001 |  |  |  |  |  |  |  |  |  |  |  |  |
| 0 | Vitvi09g02090\_t001 |  |  |  |  |  |  |  |  |  |  |  |  |
| 0 | Vitvi09g02091\_t001 |  |  |  |  |  |  |  |  |  |  |  |  |
| 0 | Vitvi09g02092\_t001 |  |  |  |  |  |  |  |  |  |  |  |  |
| 0 | Vitvi00g04210\_t001 |  |  |  |  |  |  |  |  |  |  |  |  |
| 0 | Vitvi01g02318\_t001 |  |  |  |  |  |  |  |  |  |  |  |  |
| 0 | Vitvi00g04211\_t001 |  |  |  |  |  |  |  |  |  |  |  |  |
| 0 | Vitvi00g04212\_t001 |  |  |  |  |  |  |  |  |  |  |  |  |
| 0 | Vitvi00g04213\_t001 |  |  |  |  |  |  |  |  |  |  |  |  |
| 0 | Vitvi00g04214\_t001 |  |  |  |  |  |  |  |  |  |  |  |  |
| 0 | Vitvi00g04215\_t001 |  |  |  |  |  |  |  |  |  |  |  |  |
| 0 | Vitvi00g04216\_t001 |  |  |  |  |  |  |  |  |  |  |  |  |
| 0 | Vitvi00g04217\_t001 |  |  |  |  |  |  |  |  |  |  |  |  |
| 0 | Vitvi09g02096\_t001 |  |  |  |  |  |  |  |  |  |  |  |  |
| 0 | Vitvi00g04218\_t001 |  |  |  |  |  |  |  |  |  |  |  |  |
| 0 | Vitvi00g04219\_t001 |  |  |  |  |  |  |  |  |  |  |  |  |
| 0 | Vitvi00g04220\_t001 |  |  |  |  |  |  |  |  |  |  |  |  |
| 0 | Vitvi09g02103\_t001 |  |  |  |  |  |  |  |  |  |  |  |  |
| 0 | Vitvi09g02104\_t001 |  |  |  |  |  |  |  |  |  |  |  |  |
| 0 | Vitvi00g04221\_t001 |  |  |  |  |  |  |  |  |  |  |  |  |
| 0 | Vitvi00g04222\_t001 |  |  |  |  |  |  |  |  |  |  |  |  |
| 0 | Vitvi00g04223\_t001 |  |  |  |  |  |  |  |  |  |  |  |  |
| 0 | Vitvi00g04224\_t001 |  |  |  |  |  |  |  |  |  |  |  |  |
| 0 | Vitvi09g02107\_t001 |  |  |  |  |  |  |  |  |  |  |  |  |
| 0 | Vitvi00g04225\_t001 |  |  |  |  |  |  |  |  |  |  |  |  |
| 0 | Vitvi00g04226\_t001 |  |  |  |  |  |  |  |  |  |  |  |  |
| 0 | Vitvi00g04227\_t001 |  |  |  |  |  |  |  |  |  |  |  |  |
| 0 | Vitvi00g04228\_t001 |  |  |  |  |  |  |  |  |  |  |  |  |
| 0 | Vitvi00g00973\_t001 |  |  |  |  |  |  |  |  |  |  |  |  |
| 0 | Vitvi00g00974\_t001 |  |  |  |  |  |  |  |  |  |  |  |  |
| 0 | Vitvi00g04229\_t001 |  |  |  |  |  |  |  |  |  |  |  |  |
| 0 | Vitvi00g04230\_t001 |  |  |  |  |  |  |  |  |  |  |  |  |
| 0 | Vitvi00g04231\_t001 |  |  |  |  |  |  |  |  |  |  |  |  |
| 0 | Vitvi00g01462\_t001 |  |  |  |  |  |  |  |  |  |  |  |  |
| 0 | Vitvi00g04232\_t001 |  |  |  |  |  |  |  |  |  |  |  |  |
| 0 | Vitvi00g01453\_t001 |  |  |  |  |  |  |  |  |  |  |  |  |
| 0 | Vitvi00g04233\_t001 |  |  |  |  |  |  |  |  |  |  |  |  |
| 0 | Vitvi00g04234\_t001 |  |  |  |  |  |  |  |  |  |  |  |  |
| 0 | Vitvi00g04235\_t001 |  |  |  |  |  |  |  |  |  |  |  |  |
| 0 | Vitvi00g04236\_t001 |  |  |  |  |  |  |  |  |  |  |  |  |
| 0 | Vitvi00g04237\_t001 |  |  |  |  |  |  |  |  |  |  |  |  |
| 0 | Vitvi00g04238\_t001 |  |  |  |  |  |  |  |  |  |  |  |  |
| 0 | Vitvi00g04239\_t001 |  |  |  |  |  |  |  |  |  |  |  |  |
| 0 | Vitvi00g04240\_t001 |  |  |  |  |  |  |  |  |  |  |  |  |
| 0 | Vitvi00g04241\_t001 |  |  |  |  |  |  |  |  |  |  |  |  |
| 0 | Vitvi07g02871\_t001 |  |  |  |  |  |  |  |  |  |  |  |  |
| 0 | Vitvi00g04242\_t001 |  |  |  |  |  |  |  |  |  |  |  |  |
| 0 | Vitvi00g04243\_t001 |  |  |  |  |  |  |  |  |  |  |  |  |
| 0 | Vitvi00g04244\_t001 |  |  |  |  |  |  |  |  |  |  |  |  |
| 0 | Vitvi00g04245\_t001 |  |  |  |  |  |  |  |  |  |  |  |  |
| 0 | Vitvi00g04246\_t001 |  |  |  |  |  |  |  |  |  |  |  |  |
| 0 | Vitvi00g04247\_t001 |  |  |  |  |  |  |  |  |  |  |  |  |
| 0 | Vitvi10g02353\_t001 |  |  |  |  |  |  |  |  |  |  |  |  |
| 0 | Vitvi00g04248\_t001 |  |  |  |  |  |  |  |  |  |  |  |  |
| 0 | Vitvi00g04249\_t001 |  |  |  |  |  |  |  |  |  |  |  |  |
| 0 | Vitvi00g04250\_t001 |  |  |  |  |  |  |  |  |  |  |  |  |
| 0 | Vitvi10g02358\_t001 |  |  |  |  |  |  |  |  |  |  |  |  |
| 0 | Vitvi00g04251\_t001 |  |  |  |  |  |  |  |  |  |  |  |  |
| 0 | Vitvi00g04252\_t001 |  |  |  |  |  |  |  |  |  |  |  |  |
| 0 | Vitvi00g04253\_t001 |  |  |  |  |  |  |  |  |  |  |  |  |
| 0 | Vitvi10g02362\_t001 |  |  |  |  |  |  |  |  |  |  |  |  |
| 0 | Vitvi17g01696\_t001 |  |  |  |  |  |  |  |  |  |  |  |  |
| 0 | Vitvi17g01697\_t001 |  |  |  |  |  |  |  |  |  |  |  |  |
| 0 | Vitvi00g04254\_t001 |  |  |  |  |  |  |  |  |  |  |  |  |
| 0 | Vitvi00g04255\_t001 |  |  |  |  |  |  |  |  |  |  |  |  |
| 0 | Vitvi00g00444\_t001 |  |  |  |  |  |  |  |  |  |  |  |  |
| 0 | Vitvi00g00447\_t001 |  |  |  |  |  |  |  |  |  |  |  |  |
| 0 | Vitvi00g04256\_t001 |  |  |  |  |  |  |  |  |  |  |  |  |
| 0 | Vitvi00g00449\_t001 |  |  |  |  |  |  |  |  |  |  |  |  |
| 0 | Vitvi00g04257\_t001 |  |  |  |  |  |  |  |  |  |  |  |  |
| 0 | Vitvi00g04258\_t001 |  |  |  |  |  |  |  |  |  |  |  |  |
| 0 | Vitvi00g04259\_t001 |  |  |  |  |  |  |  |  |  |  |  |  |
| 0 | Vitvi00g04260\_t001 |  |  |  |  |  |  |  |  |  |  |  |  |
| 0 | Vitvi00g04261\_t001 |  |  |  |  |  |  |  |  |  |  |  |  |
| 0 | Vitvi00g04262\_t001 |  |  |  |  |  |  |  |  |  |  |  |  |
| 0 | Vitvi00g04263\_t001 |  |  |  |  |  |  |  |  |  |  |  |  |
| 0 | Vitvi00g04264\_t001 |  |  |  |  |  |  |  |  |  |  |  |  |
| 0 | Vitvi00g04265\_t001 |  |  |  |  |  |  |  |  |  |  |  |  |
| 0 | Vitvi00g04266\_t001 |  |  |  |  |  |  |  |  |  |  |  |  |
| 0 | Vitvi00g04267\_t001 |  |  |  |  |  |  |  |  |  |  |  |  |
| 0 | Vitvi00g04268\_t001 |  |  |  |  |  |  |  |  |  |  |  |  |
| 0 | Vitvi00g04269\_t001 |  |  |  |  |  |  |  |  |  |  |  |  |
| 0 | Vitvi00g04270\_t001 |  |  |  |  |  |  |  |  |  |  |  |  |
| 0 | Vitvi00g04271\_t001 |  |  |  |  |  |  |  |  |  |  |  |  |
| 0 | Vitvi00g04272\_t001 |  |  |  |  |  |  |  |  |  |  |  |  |
| 1 | Vitvi00g04273\_t001 |  | Vitvi07g01381\_t001 |  |  |  |  |  |  |  |  |  |  |
| 1 | Vitvi00g04274\_t002 |  | Vitvi07g02548\_t001 |  |  |  |  |  |  |  |  |  |  |
| 1 | Vitvi00g04275\_t001 |  | Vitvi07g01380\_t001 |  |  |  |  |  |  |  |  |  |  |
| 1 | Vitvi00g04276\_t001 |  | \| \| |  |  |  |  |  |  |  |  |  |  |
| 1 | Vitvi00g04277\_t001 |  | \| \| |  |  |  |  |  |  |  |  |  |  |
| 1 | Vitvi00g04278\_t001 |  | \| \| |  |  |  |  |  |  |  |  |  |  |
| 1 | Vitvi00g04279\_t001 |  | Vitvi07g04554\_t001 |  |  |  |  |  |  |  |  |  |  |
| 1 | Vitvi00g04280\_t001 |  | Vitvi07g04553\_t001 |  |  |  |  |  |  |  |  |  |  |
| 1 | Vitvi00g04281\_t001 |  | \| \| |  |  |  |  |  |  |  |  |  |  |
| 1 | Vitvi00g04282\_t001 |  | Vitvi07g04552\_t001 |  |  |  |  |  |  |  |  |  |  |
| 1 | Vitvi00g04283\_t001 |  | Vitvi07g04551\_t001 |  |  |  |  |  |  |  |  |  |  |
| 0 | Vitvi00g04284\_t001 |  |  |  |  |  |  |  |  |  |  |  |  |
| 0 | Vitvi02g01819\_t001 |  |  |  |  |  |  |  |  |  |  |  |  |
| 0 | Vitvi02g01818\_t001 |  |  |  |  |  |  |  |  |  |  |  |  |
| 0 | Vitvi00g04285\_t001 |  |  |  |  |  |  |  |  |  |  |  |  |
| 0 | Vitvi00g04286\_t001 |  |  |  |  |  |  |  |  |  |  |  |  |
| 0 | Vitvi00g04287\_t001 |  |  |  |  |  |  |  |  |  |  |  |  |
| 0 | Vitvi09g02081\_t001 |  |  |  |  |  |  |  |  |  |  |  |  |
| 0 | Vitvi00g04288\_t001 |  |  |  |  |  |  |  |  |  |  |  |  |
| 0 | Vitvi00g04289\_t001 |  |  |  |  |  |  |  |  |  |  |  |  |
| 0 | Vitvi00g04290\_t001 |  |  |  |  |  |  |  |  |  |  |  |  |
| 0 | Vitvi00g04291\_t001 |  |  |  |  |  |  |  |  |  |  |  |  |
| 0 | Vitvi00g04292\_t001 |  |  |  |  |  |  |  |  |  |  |  |  |
| 0 | Vitvi00g04293\_t001 |  |  |  |  |  |  |  |  |  |  |  |  |
| 0 | Vitvi00g04294\_t001 |  |  |  |  |  |  |  |  |  |  |  |  |
| 0 | Vitvi09g02087\_t001 |  |  |  |  |  |  |  |  |  |  |  |  |
| 0 | Vitvi00g04295\_t001 |  |  |  |  |  |  |  |  |  |  |  |  |
| 0 | Vitvi00g04296\_t001 |  |  |  |  |  |  |  |  |  |  |  |  |
| 0 | Vitvi00g04297\_t001 |  |  |  |  |  |  |  |  |  |  |  |  |
| 0 | Vitvi00g04298\_t001 |  |  |  |  |  |  |  |  |  |  |  |  |
| 0 | Vitvi00g04299\_t001 |  |  |  |  |  |  |  |  |  |  |  |  |
| 0 | Vitvi00g04300\_t001 |  |  |  |  |  |  |  |  |  |  |  |  |
| 0 | Vitvi00g04301\_t001 |  |  |  |  |  |  |  |  |  |  |  |  |
| 0 | Vitvi00g04302\_t001 |  |  |  |  |  |  |  |  |  |  |  |  |
| 0 | Vitvi00g04303\_t001 |  |  |  |  |  |  |  |  |  |  |  |  |
| 0 | Vitvi00g04304\_t001 |  |  |  |  |  |  |  |  |  |  |  |  |
| 0 | Vitvi00g04305\_t001 |  |  |  |  |  |  |  |  |  |  |  |  |
| 0 | Vitvi00g01805\_t001 |  |  |  |  |  |  |  |  |  |  |  |  |
| 0 | Vitvi00g04306\_t001 |  |  |  |  |  |  |  |  |  |  |  |  |
| 0 | Vitvi00g04307\_t001 |  |  |  |  |  |  |  |  |  |  |  |  |
| 0 | Vitvi00g04308\_t001 |  |  |  |  |  |  |  |  |  |  |  |  |
| 0 | Vitvi00g00544\_t001 |  |  |  |  |  |  |  |  |  |  |  |  |
| 0 | Vitvi00g00545\_t001 |  |  |  |  |  |  |  |  |  |  |  |  |
| 0 | Vitvi00g04309\_t001 |  |  |  |  |  |  |  |  |  |  |  |  |
| 0 | Vitvi00g04310\_t001 |  |  |  |  |  |  |  |  |  |  |  |  |
| 0 | Vitvi00g04311\_t001 |  |  |  |  |  |  |  |  |  |  |  |  |
| 0 | Vitvi00g04312\_t001 |  |  |  |  |  |  |  |  |  |  |  |  |
| 0 | Vitvi00g04313\_t001 |  |  |  |  |  |  |  |  |  |  |  |  |
| 0 | Vitvi00g04314\_t001 |  |  |  |  |  |  |  |  |  |  |  |  |
| 0 | Vitvi00g04315\_t001 |  |  |  |  |  |  |  |  |  |  |  |  |
| 0 | Vitvi00g04316\_t001 |  |  |  |  |  |  |  |  |  |  |  |  |
| 0 | Vitvi00g04317\_t001 |  |  |  |  |  |  |  |  |  |  |  |  |
| 0 | Vitvi00g04318\_t001 |  |  |  |  |  |  |  |  |  |  |  |  |
| 0 | Vitvi00g00610\_t001 |  |  |  |  |  |  |  |  |  |  |  |  |
| 0 | Vitvi00g04319\_t001 |  |  |  |  |  |  |  |  |  |  |  |  |
| 0 | Vitvi00g04320\_t001 |  |  |  |  |  |  |  |  |  |  |  |  |
| 0 | Vitvi00g04321\_t001 |  |  |  |  |  |  |  |  |  |  |  |  |
| 0 | Vitvi00g04322\_t001 |  |  |  |  |  |  |  |  |  |  |  |  |
| 0 | Vitvi00g04323\_t001 |  |  |  |  |  |  |  |  |  |  |  |  |
| 0 | Vitvi00g04324\_t001 |  |  |  |  |  |  |  |  |  |  |  |  |
| 0 | Vitvi00g04325\_t001 |  |  |  |  |  |  |  |  |  |  |  |  |
| 0 | Vitvi00g00614\_t001 |  |  |  |  |  |  |  |  |  |  |  |  |
| 0 | Vitvi00g00615\_t001 |  |  |  |  |  |  |  |  |  |  |  |  |
| 0 | Vitvi00g04326\_t001 |  |  |  |  |  |  |  |  |  |  |  |  |
| 0 | Vitvi00g04327\_t001 |  |  |  |  |  |  |  |  |  |  |  |  |
| 0 | Vitvi00g04328\_t001 |  |  |  |  |  |  |  |  |  |  |  |  |
| 0 | Vitvi00g04329\_t001 |  |  |  |  |  |  |  |  |  |  |  |  |
| 0 | Vitvi00g04330\_t001 |  |  |  |  |  |  |  |  |  |  |  |  |
| 0 | Vitvi00g04331\_t001 |  |  |  |  |  |  |  |  |  |  |  |  |
| 0 | Vitvi00g04332\_t001 |  |  |  |  |  |  |  |  |  |  |  |  |
| 1 | Vitvi00g04333\_t001 |  | Vitvi02g00454\_t001 |  |  |  |  |  |  |  |  |  |  |
| 1 | Vitvi00g04334\_t001 |  | Vitvi02g04115\_t001 |  |  |  |  |  |  |  |  |  |  |
| 1 | Vitvi00g04335\_t001 |  | Vitvi02g04116\_t001 |  |  |  |  |  |  |  |  |  |  |
| 1 | Vitvi00g04336\_t001 |  | Vitvi02g04117\_t001 |  |  |  |  |  |  |  |  |  |  |
| 1 | Vitvi00g04337\_t001 |  | Vitvi02g04118\_t001 |  |  |  |  |  |  |  |  |  |  |
| 1 | Vitvi00g04338\_t001 |  | Vitvi02g04119\_t001 |  |  |  |  |  |  |  |  |  |  |
| 1 | Vitvi00g04339\_t001 |  | Vitvi02g04120\_t001 |  |  |  |  |  |  |  |  |  |  |
| 1 | Vitvi00g04340\_t001 |  | Vitvi02g00459\_t001 |  |  |  |  |  |  |  |  |  |  |
| 0 | Vitvi00g04341\_t001 |  |  |  |  |  |  |  |  |  |  |  |  |
| 0 | Vitvi00g04342\_t001 |  |  |  |  |  |  |  |  |  |  |  |  |
| 0 | Vitvi00g04343\_t001 |  |  |  |  |  |  |  |  |  |  |  |  |
| 0 | Vitvi00g04344\_t001 |  |  |  |  |  |  |  |  |  |  |  |  |
| 0 | Vitvi00g04345\_t001 |  |  |  |  |  |  |  |  |  |  |  |  |
| 0 | Vitvi00g04346\_t001 |  |  |  |  |  |  |  |  |  |  |  |  |
| 0 | Vitvi00g04347\_t001 |  |  |  |  |  |  |  |  |  |  |  |  |
| 0 | Vitvi00g04348\_t001 |  |  |  |  |  |  |  |  |  |  |  |  |
| 0 | Vitvi00g04349\_t001 |  |  |  |  |  |  |  |  |  |  |  |  |
| 0 | Vitvi00g04350\_t001 |  |  |  |  |  |  |  |  |  |  |  |  |
| 0 | Vitvi00g04351\_t001 |  |  |  |  |  |  |  |  |  |  |  |  |
| 0 | Vitvi00g04352\_t001 |  |  |  |  |  |  |  |  |  |  |  |  |
| 0 | Vitvi00g04353\_t001 |  |  |  |  |  |  |  |  |  |  |  |  |
| 0 | Vitvi00g04354\_t001 |  |  |  |  |  |  |  |  |  |  |  |  |
| 0 | Vitvi00g04355\_t001 |  |  |  |  |  |  |  |  |  |  |  |  |
| 0 | Vitvi00g04356\_t001 |  |  |  |  |  |  |  |  |  |  |  |  |
| 0 | Vitvi00g04357\_t001 |  |  |  |  |  |  |  |  |  |  |  |  |
| 0 | Vitvi00g04358\_t001 |  |  |  |  |  |  |  |  |  |  |  |  |
| 0 | Vitvi00g04359\_t001 |  |  |  |  |  |  |  |  |  |  |  |  |
| 0 | Vitvi00g04360\_t001 |  |  |  |  |  |  |  |  |  |  |  |  |
| 0 | Vitvi00g04361\_t001 |  |  |  |  |  |  |  |  |  |  |  |  |
| 0 | Vitvi00g04362\_t001 |  |  |  |  |  |  |  |  |  |  |  |  |
| 0 | Vitvi00g04363\_t001 |  |  |  |  |  |  |  |  |  |  |  |  |
| 0 | Vitvi00g04364\_t001 |  |  |  |  |  |  |  |  |  |  |  |  |
| 0 | Vitvi00g04365\_t001 |  |  |  |  |  |  |  |  |  |  |  |  |
| 0 | Vitvi00g04366\_t001 |  |  |  |  |  |  |  |  |  |  |  |  |
| 0 | Vitvi00g04367\_t001 |  |  |  |  |  |  |  |  |  |  |  |  |
| 0 | Vitvi00g04368\_t001 |  |  |  |  |  |  |  |  |  |  |  |  |
| 0 | Vitvi00g04369\_t001 |  |  |  |  |  |  |  |  |  |  |  |  |
| 0 | Vitvi00g04370\_t001 |  |  |  |  |  |  |  |  |  |  |  |  |
| 0 | Vitvi00g04371\_t001 |  |  |  |  |  |  |  |  |  |  |  |  |
| 0 | Vitvi00g04372\_t001 |  |  |  |  |  |  |  |  |  |  |  |  |
| 0 | Vitvi00g04373\_t001 |  |  |  |  |  |  |  |  |  |  |  |  |
| 0 | Vitvi00g04374\_t001 |  |  |  |  |  |  |  |  |  |  |  |  |
| 0 | Vitvi00g04375\_t001 |  |  |  |  |  |  |  |  |  |  |  |  |
| 0 | Vitvi00g04376\_t001 |  |  |  |  |  |  |  |  |  |  |  |  |
| 0 | Vitvi00g04377\_t001 |  |  |  |  |  |  |  |  |  |  |  |  |
| 0 | Vitvi00g04378\_t001 |  |  |  |  |  |  |  |  |  |  |  |  |
| 0 | Vitvi00g04379\_t001 |  |  |  |  |  |  |  |  |  |  |  |  |
| 0 | Vitvi00g04380\_t001 |  |  |  |  |  |  |  |  |  |  |  |  |
| 0 | Vitvi00g04381\_t001 |  |  |  |  |  |  |  |  |  |  |  |  |
| 0 | Vitvi00g04382\_t001 |  |  |  |  |  |  |  |  |  |  |  |  |
| 0 | Vitvi00g04383\_t001 |  |  |  |  |  |  |  |  |  |  |  |  |
| 0 | Vitvi00g04384\_t001 |  |  |  |  |  |  |  |  |  |  |  |  |
| 0 | Vitvi00g04385\_t001 |  |  |  |  |  |  |  |  |  |  |  |  |
| 0 | Vitvi00g04386\_t001 |  |  |  |  |  |  |  |  |  |  |  |  |
| 0 | Vitvi00g04387\_t001 |  |  |  |  |  |  |  |  |  |  |  |  |
| 0 | Vitvi00g04388\_t001 |  |  |  |  |  |  |  |  |  |  |  |  |
| 0 | Vitvi00g04389\_t001 |  |  |  |  |  |  |  |  |  |  |  |  |
| 0 | Vitvi00g04390\_t001 |  |  |  |  |  |  |  |  |  |  |  |  |
| 0 | Vitvi00g04391\_t001 |  |  |  |  |  |  |  |  |  |  |  |  |
| 0 | Vitvi00g04392\_t001 |  |  |  |  |  |  |  |  |  |  |  |  |
| 0 | Vitvi00g04393\_t001 |  |  |  |  |  |  |  |  |  |  |  |  |
| 0 | Vitvi00g04394\_t001 |  |  |  |  |  |  |  |  |  |  |  |  |
| 0 | Vitvi00g04395\_t001 |  |  |  |  |  |  |  |  |  |  |  |  |
| 0 | Vitvi00g04396\_t001 |  |  |  |  |  |  |  |  |  |  |  |  |
| 0 | Vitvi00g04397\_t001 |  |  |  |  |  |  |  |  |  |  |  |  |
| 0 | Vitvi00g04398\_t001 |  |  |  |  |  |  |  |  |  |  |  |  |
| 0 | Vitvi00g04399\_t001 |  |  |  |  |  |  |  |  |  |  |  |  |
| 0 | Vitvi00g04400\_t001 |  |  |  |  |  |  |  |  |  |  |  |  |
| 0 | Vitvi00g04401\_t001 |  |  |  |  |  |  |  |  |  |  |  |  |
| 0 | Vitvi00g04402\_t001 |  |  |  |  |  |  |  |  |  |  |  |  |
| 0 | Vitvi00g04404\_t001 |  |  |  |  |  |  |  |  |  |  |  |  |
| 0 | Vitvi00g04405\_t001 |  |  |  |  |  |  |  |  |  |  |  |  |
| 0 | Vitvi00g04406\_t001 |  |  |  |  |  |  |  |  |  |  |  |  |
| 0 | Vitvi00g04407\_t001 |  |  |  |  |  |  |  |  |  |  |  |  |
| 0 | Vitvi00g04408\_t001 |  |  |  |  |  |  |  |  |  |  |  |  |
| 0 | Vitvi00g04409\_t001 |  |  |  |  |  |  |  |  |  |  |  |  |
| 0 | Vitvi00g04410\_t001 |  |  |  |  |  |  |  |  |  |  |  |  |
| 0 | Vitvi00g04411\_t001 |  |  |  |  |  |  |  |  |  |  |  |  |
| 0 | Vitvi00g04412\_t001 |  |  |  |  |  |  |  |  |  |  |  |  |
| 0 | Vitvi00g04413\_t001 |  |  |  |  |  |  |  |  |  |  |  |  |
| 0 | Vitvi00g04414\_t001 |  |  |  |  |  |  |  |  |  |  |  |  |
| 0 | Vitvi00g04415\_t001 |  |  |  |  |  |  |  |  |  |  |  |  |
| 0 | Vitvi00g04416\_t001 |  |  |  |  |  |  |  |  |  |  |  |  |
| 0 | Vitvi00g04417\_t001 |  |  |  |  |  |  |  |  |  |  |  |  |
| 0 | Vitvi00g04418\_t001 |  |  |  |  |  |  |  |  |  |  |  |  |
| 0 | Vitvi00g04419\_t001 |  |  |  |  |  |  |  |  |  |  |  |  |
| 0 | Vitvi00g04420\_t001 |  |  |  |  |  |  |  |  |  |  |  |  |
| 0 | Vitvi00g04421\_t001 |  |  |  |  |  |  |  |  |  |  |  |  |
| 0 | Vitvi00g04422\_t001 |  |  |  |  |  |  |  |  |  |  |  |  |
| 0 | Vitvi00g04423\_t001 |  |  |  |  |  |  |  |  |  |  |  |  |
| 0 | Vitvi00g04424\_t001 |  |  |  |  |  |  |  |  |  |  |  |  |
| 0 | Vitvi00g04425\_t001 |  |  |  |  |  |  |  |  |  |  |  |  |
| 0 | Vitvi00g04426\_t001 |  |  |  |  |  |  |  |  |  |  |  |  |
| 0 | Vitvi00g04427\_t001 |  |  |  |  |  |  |  |  |  |  |  |  |
| 0 | Vitvi00g04428\_t001 |  |  |  |  |  |  |  |  |  |  |  |  |
| 0 | Vitvi00g04429\_t001 |  |  |  |  |  |  |  |  |  |  |  |  |
| 0 | Vitvi00g04430\_t001 |  |  |  |  |  |  |  |  |  |  |  |  |
| 0 | Vitvi00g04431\_t001 |  |  |  |  |  |  |  |  |  |  |  |  |
| 0 | Vitvi00g04432\_t001 |  |  |  |  |  |  |  |  |  |  |  |  |
| 0 | Vitvi00g04433\_t001 |  |  |  |  |  |  |  |  |  |  |  |  |
| 0 | Vitvi09g02052\_t001 |  |  |  |  |  |  |  |  |  |  |  |  |
| 0 | Vitvi00g04434\_t001 |  |  |  |  |  |  |  |  |  |  |  |  |
| 0 | Vitvi00g04435\_t001 |  |  |  |  |  |  |  |  |  |  |  |  |
| 0 | Vitvi00g04436\_t001 |  |  |  |  |  |  |  |  |  |  |  |  |
| 0 | Vitvi00g04437\_t001 |  |  |  |  |  |  |  |  |  |  |  |  |
| 0 | Vitvi00g04438\_t001 |  |  |  |  |  |  |  |  |  |  |  |  |
| 0 | Vitvi00g04439\_t001 |  |  |  |  |  |  |  |  |  |  |  |  |
| 0 | Vitvi00g04440\_t001 |  |  |  |  |  |  |  |  |  |  |  |  |
| 0 | Vitvi00g04441\_t001 |  |  |  |  |  |  |  |  |  |  |  |  |
| 0 | Vitvi00g04442\_t001 |  |  |  |  |  |  |  |  |  |  |  |  |
| 0 | Vitvi00g04443\_t001 |  |  |  |  |  |  |  |  |  |  |  |  |
| 0 | Vitvi00g04444\_t001 |  |  |  |  |  |  |  |  |  |  |  |  |
| 0 | Vitvi00g04445\_t001 |  |  |  |  |  |  |  |  |  |  |  |  |
| 0 | Vitvi00g04446\_t001 |  |  |  |  |  |  |  |  |  |  |  |  |
| 0 | Vitvi00g04447\_t001 |  |  |  |  |  |  |  |  |  |  |  |  |
| 0 | Vitvi00g04448\_t001 |  |  |  |  |  |  |  |  |  |  |  |  |
| 0 | Vitvi00g04449\_t001 |  |  |  |  |  |  |  |  |  |  |  |  |
| 0 | Vitvi00g04450\_t001 |  |  |  |  |  |  |  |  |  |  |  |  |
| 0 | Vitvi00g04451\_t001 |  |  |  |  |  |  |  |  |  |  |  |  |
| 0 | Vitvi00g04452\_t001 |  |  |  |  |  |  |  |  |  |  |  |  |
| 0 | Vitvi00g04453\_t001 |  |  |  |  |  |  |  |  |  |  |  |  |
| 0 | Vitvi00g04454\_t001 |  |  |  |  |  |  |  |  |  |  |  |  |
| 0 | Vitvi00g04455\_t001 |  |  |  |  |  |  |  |  |  |  |  |  |
| 0 | Vitvi00g04456\_t001 |  |  |  |  |  |  |  |  |  |  |  |  |
| 0 | Vitvi00g04457\_t001 |  |  |  |  |  |  |  |  |  |  |  |  |
| 0 | Vitvi00g04458\_t001 |  |  |  |  |  |  |  |  |  |  |  |  |
| 0 | Vitvi00g04459\_t001 |  |  |  |  |  |  |  |  |  |  |  |  |
| 0 | Vitvi00g04460\_t001 |  |  |  |  |  |  |  |  |  |  |  |  |
| 0 | Vitvi00g04461\_t001 |  |  |  |  |  |  |  |  |  |  |  |  |
| 0 | Vitvi00g04462\_t001 |  |  |  |  |  |  |  |  |  |  |  |  |
| 0 | Vitvi07g03105\_t001 |  |  |  |  |  |  |  |  |  |  |  |  |
| 0 | Vitvi00g04463\_t001 |  |  |  |  |  |  |  |  |  |  |  |  |
| 0 | Vitvi00g04464\_t001 |  |  |  |  |  |  |  |  |  |  |  |  |
| 0 | Vitvi00g04465\_t001 |  |  |  |  |  |  |  |  |  |  |  |  |
| 0 | Vitvi00g04466\_t001 |  |  |  |  |  |  |  |  |  |  |  |  |
| 0 | Vitvi00g04467\_t001 |  |  |  |  |  |  |  |  |  |  |  |  |
| 0 | Vitvi00g04468\_t001 |  |  |  |  |  |  |  |  |  |  |  |  |
| 0 | Vitvi00g04469\_t001 |  |  |  |  |  |  |  |  |  |  |  |  |
| 0 | Vitvi00g04470\_t001 |  |  |  |  |  |  |  |  |  |  |  |  |
| 0 | Vitvi00g04471\_t001 |  |  |  |  |  |  |  |  |  |  |  |  |
| 0 | Vitvi07g02803\_t002 |  |  |  |  |  |  |  |  |  |  |  |  |
| 0 | Vitvi00g04472\_t001 |  |  |  |  |  |  |  |  |  |  |  |  |
| 0 | Vitvi00g04473\_t001 |  |  |  |  |  |  |  |  |  |  |  |  |
| 0 | Vitvi00g04474\_t001 |  |  |  |  |  |  |  |  |  |  |  |  |
| 0 | Vitvi00g04475\_t001 |  |  |  |  |  |  |  |  |  |  |  |  |
| 0 | Vitvi00g04476\_t001 |  |  |  |  |  |  |  |  |  |  |  |  |
| 0 | Vitvi00g04477\_t001 |  |  |  |  |  |  |  |  |  |  |  |  |
| 0 | Vitvi00g04478\_t001 |  |  |  |  |  |  |  |  |  |  |  |  |
| 0 | Vitvi00g04479\_t001 |  |  |  |  |  |  |  |  |  |  |  |  |
| 0 | Vitvi00g04480\_t001 |  |  |  |  |  |  |  |  |  |  |  |  |
| 0 | Vitvi00g04481\_t001 |  |  |  |  |  |  |  |  |  |  |  |  |
| 0 | Vitvi02g01817\_t001 |  |  |  |  |  |  |  |  |  |  |  |  |
| 0 | Vitvi02g01816\_t001 |  |  |  |  |  |  |  |  |  |  |  |  |
| 0 | Vitvi02g01815\_t001 |  |  |  |  |  |  |  |  |  |  |  |  |
| 0 | Vitvi02g01813\_t001 |  |  |  |  |  |  |  |  |  |  |  |  |
| 0 | Vitvi02g01814\_t001 |  |  |  |  |  |  |  |  |  |  |  |  |
| 0 | Vitvi00g04482\_t001 |  |  |  |  |  |  |  |  |  |  |  |  |
| 0 | Vitvi00g04483\_t001 |  |  |  |  |  |  |  |  |  |  |  |  |
| 0 | Vitvi00g04484\_t001 |  |  |  |  |  |  |  |  |  |  |  |  |
| 0 | Vitvi00g04485\_t001 |  |  |  |  |  |  |  |  |  |  |  |  |
| 0 | Vitvi00g04486\_t001 |  |  |  |  |  |  |  |  |  |  |  |  |
| 0 | Vitvi00g04487\_t001 |  |  |  |  |  |  |  |  |  |  |  |  |
| 0 | Vitvi00g04488\_t001 |  |  |  |  |  |  |  |  |  |  |  |  |
| 0 | Vitvi00g04489\_t002 |  |  |  |  |  |  |  |  |  |  |  |  |
| 0 | Vitvi00g04490\_t001 |  |  |  |  |  |  |  |  |  |  |  |  |
| 0 | Vitvi00g04491\_t001 |  |  |  |  |  |  |  |  |  |  |  |  |
| 0 | Vitvi00g04492\_t001 |  |  |  |  |  |  |  |  |  |  |  |  |
| 0 | Vitvi00g04493\_t001 |  |  |  |  |  |  |  |  |  |  |  |  |
| 0 | Vitvi00g04494\_t001 |  |  |  |  |  |  |  |  |  |  |  |  |
| 0 | Vitvi00g04495\_t001 |  |  |  |  |  |  |  |  |  |  |  |  |
| 0 | Vitvi00g04496\_t001 |  |  |  |  |  |  |  |  |  |  |  |  |
| 0 | Vitvi00g04497\_t001 |  |  |  |  |  |  |  |  |  |  |  |  |
| 0 | Vitvi00g04498\_t001 |  |  |  |  |  |  |  |  |  |  |  |  |
| 0 | Vitvi00g04499\_t001 |  |  |  |  |  |  |  |  |  |  |  |  |
| 0 | Vitvi00g04500\_t001 |  |  |  |  |  |  |  |  |  |  |  |  |
| 0 | Vitvi00g04501\_t001 |  |  |  |  |  |  |  |  |  |  |  |  |
| 0 | Vitvi00g04502\_t001 |  |  |  |  |  |  |  |  |  |  |  |  |
| 0 | Vitvi00g04503\_t001 |  |  |  |  |  |  |  |  |  |  |  |  |
| 0 | Vitvi00g04504\_t001 |  |  |  |  |  |  |  |  |  |  |  |  |
| 0 | Vitvi00g04505\_t002 |  |  |  |  |  |  |  |  |  |  |  |  |
| 0 | Vitvi00g04506\_t001 |  |  |  |  |  |  |  |  |  |  |  |  |
| 0 | Vitvi00g04507\_t002 |  |  |  |  |  |  |  |  |  |  |  |  |
| 0 | Vitvi00g04508\_t001 |  |  |  |  |  |  |  |  |  |  |  |  |
| 0 | Vitvi00g04509\_t001 |  |  |  |  |  |  |  |  |  |  |  |  |
| 0 | Vitvi00g04510\_t001 |  |  |  |  |  |  |  |  |  |  |  |  |
| 0 | Vitvi00g04511\_t001 |  |  |  |  |  |  |  |  |  |  |  |  |
| 0 | Vitvi00g04512\_t001 |  |  |  |  |  |  |  |  |  |  |  |  |
| 0 | Vitvi00g04513\_t001 |  |  |  |  |  |  |  |  |  |  |  |  |
| 0 | Vitvi00g04514\_t001 |  |  |  |  |  |  |  |  |  |  |  |  |
| 0 | Vitvi00g04515\_t001 |  |  |  |  |  |  |  |  |  |  |  |  |
| 0 | Vitvi00g04516\_t001 |  |  |  |  |  |  |  |  |  |  |  |  |
| 0 | Vitvi00g04517\_t001 |  |  |  |  |  |  |  |  |  |  |  |  |
| 0 | Vitvi00g04518\_t001 |  |  |  |  |  |  |  |  |  |  |  |  |
| 0 | Vitvi00g04519\_t001 |  |  |  |  |  |  |  |  |  |  |  |  |
| 0 | Vitvi00g04520\_t001 |  |  |  |  |  |  |  |  |  |  |  |  |
| 0 | Vitvi00g04521\_t001 |  |  |  |  |  |  |  |  |  |  |  |  |
| 0 | Vitvi00g04522\_t001 |  |  |  |  |  |  |  |  |  |  |  |  |
| 0 | Vitvi00g04523\_t001 |  |  |  |  |  |  |  |  |  |  |  |  |
| 0 | Vitvi00g04524\_t001 |  |  |  |  |  |  |  |  |  |  |  |  |
| 0 | Vitvi00g04525\_t001 |  |  |  |  |  |  |  |  |  |  |  |  |
| 0 | Vitvi00g04526\_t001 |  |  |  |  |  |  |  |  |  |  |  |  |
| 0 | Vitvi00g04527\_t001 |  |  |  |  |  |  |  |  |  |  |  |  |
| 0 | Vitvi00g04528\_t001 |  |  |  |  |  |  |  |  |  |  |  |  |
| 0 | Vitvi00g00786\_t001 |  |  |  |  |  |  |  |  |  |  |  |  |
| 0 | Vitvi00g04529\_t001 |  |  |  |  |  |  |  |  |  |  |  |  |
| 0 | Vitvi00g04530\_t001 |  |  |  |  |  |  |  |  |  |  |  |  |
| 0 | Vitvi00g04531\_t001 |  |  |  |  |  |  |  |  |  |  |  |  |
| 0 | Vitvi00g04532\_t001 |  |  |  |  |  |  |  |  |  |  |  |  |
| 0 | Vitvi00g04533\_t001 |  |  |  |  |  |  |  |  |  |  |  |  |
| 0 | Vitvi00g04534\_t001 |  |  |  |  |  |  |  |  |  |  |  |  |
| 0 | Vitvi00g04535\_t001 |  |  |  |  |  |  |  |  |  |  |  |  |
| 0 | Vitvi00g04536\_t001 |  |  |  |  |  |  |  |  |  |  |  |  |
| 0 | Vitvi00g04537\_t001 |  |  |  |  |  |  |  |  |  |  |  |  |
| 0 | Vitvi00g04538\_t001 |  |  |  |  |  |  |  |  |  |  |  |  |
| 0 | Vitvi00g04539\_t001 |  |  |  |  |  |  |  |  |  |  |  |  |
| 0 | Vitvi00g04540\_t001 |  |  |  |  |  |  |  |  |  |  |  |  |
| 0 | Vitvi00g04541\_t001 |  |  |  |  |  |  |  |  |  |  |  |  |
| 0 | Vitvi00g04542\_t001 |  |  |  |  |  |  |  |  |  |  |  |  |
| 0 | Vitvi00g04543\_t001 |  |  |  |  |  |  |  |  |  |  |  |  |
| 0 | Vitvi00g04544\_t001 |  |  |  |  |  |  |  |  |  |  |  |  |
| 0 | Vitvi00g04545\_t001 |  |  |  |  |  |  |  |  |  |  |  |  |
| 0 | Vitvi00g04546\_t001 |  |  |  |  |  |  |  |  |  |  |  |  |
| 0 | Vitvi00g04547\_t001 |  |  |  |  |  |  |  |  |  |  |  |  |
| 0 | Vitvi00g04548\_t001 |  |  |  |  |  |  |  |  |  |  |  |  |
| 0 | Vitvi00g04549\_t001 |  |  |  |  |  |  |  |  |  |  |  |  |
| 0 | Vitvi00g04550\_t001 |  |  |  |  |  |  |  |  |  |  |  |  |
| 0 | Vitvi00g04551\_t001 |  |  |  |  |  |  |  |  |  |  |  |  |
| 0 | Vitvi00g04552\_t001 |  |  |  |  |  |  |  |  |  |  |  |  |
| 0 | Vitvi00g04553\_t001 |  |  |  |  |  |  |  |  |  |  |  |  |
| 0 | Vitvi00g04554\_t001 |  |  |  |  |  |  |  |  |  |  |  |  |
| 0 | Vitvi00g04555\_t001 |  |  |  |  |  |  |  |  |  |  |  |  |
| 0 | Vitvi00g04556\_t001 |  |  |  |  |  |  |  |  |  |  |  |  |
| 0 | Vitvi18g03302\_t001 |  |  |  |  |  |  |  |  |  |  |  |  |
| 0 | Vitvi07g02943\_t001 |  |  |  |  |  |  |  |  |  |  |  |  |
| 0 | Vitvi00g04557\_t001 |  |  |  |  |  |  |  |  |  |  |  |  |
| 0 | Vitvi07g02978\_t001 |  |  |  |  |  |  |  |  |  |  |  |  |
| 0 | Vitvi07g02979\_t001 |  |  |  |  |  |  |  |  |  |  |  |  |
| 0 | Vitvi00g04558\_t001 |  |  |  |  |  |  |  |  |  |  |  |  |
| 0 | Vitvi00g04559\_t001 |  |  |  |  |  |  |  |  |  |  |  |  |
| 0 | Vitvi00g04560\_t001 |  |  |  |  |  |  |  |  |  |  |  |  |
| 0 | Vitvi00g04561\_t001 |  |  |  |  |  |  |  |  |  |  |  |  |
| 0 | Vitvi00g04562\_t001 |  |  |  |  |  |  |  |  |  |  |  |  |
| 0 | Vitvi07g03085\_t001 |  |  |  |  |  |  |  |  |  |  |  |  |
| 0 | Vitvi07g03083\_t002 |  |  |  |  |  |  |  |  |  |  |  |  |
| 0 | Vitvi00g04563\_t001 |  |  |  |  |  |  |  |  |  |  |  |  |
| 0 | Vitvi00g04564\_t001 |  |  |  |  |  |  |  |  |  |  |  |  |
| 0 | Vitvi00g04565\_t001 |  |  |  |  |  |  |  |  |  |  |  |  |
| 0 | Vitvi00g04566\_t001 |  |  |  |  |  |  |  |  |  |  |  |  |
| 0 | Vitvi00g04567\_t001 |  |  |  |  |  |  |  |  |  |  |  |  |
| 0 | Vitvi00g04568\_t001 |  |  |  |  |  |  |  |  |  |  |  |  |
| 0 | Vitvi00g04569\_t001 |  |  |  |  |  |  |  |  |  |  |  |  |
| 0 | Vitvi00g04570\_t001 |  |  |  |  |  |  |  |  |  |  |  |  |
| 0 | Vitvi00g04571\_t001 |  |  |  |  |  |  |  |  |  |  |  |  |
| 0 | Vitvi00g04572\_t001 |  |  |  |  |  |  |  |  |  |  |  |  |
| 0 | Vitvi00g04573\_t001 |  |  |  |  |  |  |  |  |  |  |  |  |
| 0 | Vitvi00g04574\_t001 |  |  |  |  |  |  |  |  |  |  |  |  |
| 0 | Vitvi00g04576\_t001 |  |  |  |  |  |  |  |  |  |  |  |  |
| 0 | Vitvi00g04577\_t001 |  |  |  |  |  |  |  |  |  |  |  |  |
| 0 | Vitvi00g04578\_t001 |  |  |  |  |  |  |  |  |  |  |  |  |
| 0 | Vitvi09g02058\_t001 |  |  |  |  |  |  |  |  |  |  |  |  |
| 0 | Vitvi00g04579\_t001 |  |  |  |  |  |  |  |  |  |  |  |  |
| 0 | Vitvi00g04580\_t001 |  |  |  |  |  |  |  |  |  |  |  |  |
| 0 | Vitvi00g00859\_t001 |  |  |  |  |  |  |  |  |  |  |  |  |
| 0 | Vitvi00g04581\_t001 |  |  |  |  |  |  |  |  |  |  |  |  |
| 0 | Vitvi00g04582\_t001 |  |  |  |  |  |  |  |  |  |  |  |  |
| 0 | Vitvi00g04583\_t001 |  |  |  |  |  |  |  |  |  |  |  |  |
| 0 | Vitvi00g04584\_t001 |  |  |  |  |  |  |  |  |  |  |  |  |
| 0 | Vitvi00g04585\_t001 |  |  |  |  |  |  |  |  |  |  |  |  |
| 0 | Vitvi00g04586\_t001 |  |  |  |  |  |  |  |  |  |  |  |  |
| 0 | Vitvi00g04587\_t001 |  |  |  |  |  |  |  |  |  |  |  |  |
| 0 | Vitvi00g04588\_t001 |  |  |  |  |  |  |  |  |  |  |  |  |
| 0 | Vitvi00g04589\_t001 |  |  |  |  |  |  |  |  |  |  |  |  |
| 0 | Vitvi00g04590\_t001 |  |  |  |  |  |  |  |  |  |  |  |  |
| 0 | Vitvi00g04591\_t001 |  |  |  |  |  |  |  |  |  |  |  |  |
| 0 | Vitvi00g04592\_t001 |  |  |  |  |  |  |  |  |  |  |  |  |
| 0 | Vitvi00g04593\_t001 |  |  |  |  |  |  |  |  |  |  |  |  |
| 0 | Vitvi00g04594\_t001 |  |  |  |  |  |  |  |  |  |  |  |  |
| 0 | Vitvi00g04595\_t001 |  |  |  |  |  |  |  |  |  |  |  |  |
| 0 | Vitvi00g04596\_t001 |  |  |  |  |  |  |  |  |  |  |  |  |
| 0 | Vitvi00g04597\_t001 |  |  |  |  |  |  |  |  |  |  |  |  |
| 0 | Vitvi00g04598\_t001 |  |  |  |  |  |  |  |  |  |  |  |  |
| 0 | Vitvi00g04599\_t001 |  |  |  |  |  |  |  |  |  |  |  |  |
| 0 | Vitvi00g04600\_t001 |  |  |  |  |  |  |  |  |  |  |  |  |
| 0 | Vitvi00g04601\_t001 |  |  |  |  |  |  |  |  |  |  |  |  |
| 0 | Vitvi00g04602\_t001 |  |  |  |  |  |  |  |  |  |  |  |  |
| 0 | Vitvi00g04603\_t001 |  |  |  |  |  |  |  |  |  |  |  |  |
| 0 | Vitvi00g04604\_t001 |  |  |  |  |  |  |  |  |  |  |  |  |
| 0 | Vitvi00g04605\_t001 |  |  |  |  |  |  |  |  |  |  |  |  |
| 0 | Vitvi00g04606\_t001 |  |  |  |  |  |  |  |  |  |  |  |  |
| 0 | Vitvi00g04607\_t001 |  |  |  |  |  |  |  |  |  |  |  |  |
| 0 | Vitvi00g04608\_t001 |  |  |  |  |  |  |  |  |  |  |  |  |
| 0 | Vitvi00g04609\_t001 |  |  |  |  |  |  |  |  |  |  |  |  |
| 0 | Vitvi00g04610\_t001 |  |  |  |  |  |  |  |  |  |  |  |  |
| 0 | Vitvi00g04611\_t001 |  |  |  |  |  |  |  |  |  |  |  |  |
| 0 | Vitvi00g04612\_t001 |  |  |  |  |  |  |  |  |  |  |  |  |
| 0 | Vitvi00g04613\_t001 |  |  |  |  |  |  |  |  |  |  |  |  |
| 0 | Vitvi00g04614\_t001 |  |  |  |  |  |  |  |  |  |  |  |  |
| 0 | Vitvi00g04615\_t001 |  |  |  |  |  |  |  |  |  |  |  |  |
| 0 | Vitvi00g04616\_t001 |  |  |  |  |  |  |  |  |  |  |  |  |
| 0 | Vitvi00g04617\_t001 |  |  |  |  |  |  |  |  |  |  |  |  |
| 0 | Vitvi07g03088\_t001 |  |  |  |  |  |  |  |  |  |  |  |  |
| 0 | Vitvi00g04618\_t001 |  |  |  |  |  |  |  |  |  |  |  |  |
| 0 | Vitvi07g03086\_t001 |  |  |  |  |  |  |  |  |  |  |  |  |
| 0 | Vitvi03g01891\_t001 |  |  |  |  |  |  |  |  |  |  |  |  |
| 0 | Vitvi00g04619\_t001 |  |  |  |  |  |  |  |  |  |  |  |  |
| 0 | Vitvi00g04620\_t001 |  |  |  |  |  |  |  |  |  |  |  |  |
| 0 | Vitvi00g04621\_t001 |  |  |  |  |  |  |  |  |  |  |  |  |
| 0 | Vitvi00g04622\_t001 |  |  |  |  |  |  |  |  |  |  |  |  |
| 0 | Vitvi00g04623\_t001 |  |  |  |  |  |  |  |  |  |  |  |  |
| 0 | Vitvi00g04624\_t001 |  |  |  |  |  |  |  |  |  |  |  |  |
| 0 | Vitvi10g02289\_t001 |  |  |  |  |  |  |  |  |  |  |  |  |
| 0 | Vitvi00g04625\_t001 |  |  |  |  |  |  |  |  |  |  |  |  |
| 0 | Vitvi00g04626\_t001 |  |  |  |  |  |  |  |  |  |  |  |  |
| 0 | Vitvi00g04627\_t001 |  |  |  |  |  |  |  |  |  |  |  |  |
| 0 | Vitvi00g04628\_t001 |  |  |  |  |  |  |  |  |  |  |  |  |
| 0 | Vitvi00g04629\_t001 |  |  |  |  |  |  |  |  |  |  |  |  |
| 0 | Vitvi00g04630\_t001 |  |  |  |  |  |  |  |  |  |  |  |  |
| 0 | Vitvi00g04631\_t001 |  |  |  |  |  |  |  |  |  |  |  |  |
| 0 | Vitvi00g04632\_t001 |  |  |  |  |  |  |  |  |  |  |  |  |
| 0 | Vitvi00g04633\_t001 |  |  |  |  |  |  |  |  |  |  |  |  |
| 0 | Vitvi00g04634\_t001 |  |  |  |  |  |  |  |  |  |  |  |  |
| 0 | Vitvi00g04635\_t001 |  |  |  |  |  |  |  |  |  |  |  |  |
| 0 | Vitvi00g04636\_t001 |  |  |  |  |  |  |  |  |  |  |  |  |
| 0 | Vitvi00g04637\_t001 |  |  |  |  |  |  |  |  |  |  |  |  |
| 0 | Vitvi00g04638\_t001 |  |  |  |  |  |  |  |  |  |  |  |  |
| 1 | Vitvi00g04639\_t001 |  | Vitvi10g00232\_t001 |  |  |  |  |  |  |  |  |  |  |
| 1 | Vitvi00g04640\_t001 |  | Vitvi10g04108\_t001 |  |  |  |  |  |  |  |  |  |  |
| 1 | Vitvi00g04641\_t001 |  | Vitvi10g04109\_t001 |  |  |  |  |  |  |  |  |  |  |
| 1 | Vitvi00g04642\_t001 |  | Vitvi10g01699\_t001 |  |  |  |  |  |  |  |  |  |  |
| 1 | Vitvi00g04643\_t001 |  | \| \| |  |  |  |  |  |  |  |  |  |  |
| 1 | Vitvi00g04644\_t001 |  | \| \| |  |  |  |  |  |  |  |  |  |  |
| 1 | Vitvi00g04645\_t001 |  | \| \| |  |  |  |  |  |  |  |  |  |  |
| 1 | Vitvi00g04646\_t001 |  | \| \| |  |  |  |  |  |  |  |  |  |  |
| 1 | Vitvi00g04647\_t001 |  | \| \| |  |  |  |  |  |  |  |  |  |  |
| 1 | Vitvi00g04648\_t001 |  | \| \| |  |  |  |  |  |  |  |  |  |  |
| 1 | Vitvi00g04649\_t001 |  | \| \| |  |  |  |  |  |  |  |  |  |  |
| 1 | Vitvi00g04650\_t001 |  | \| \| |  |  |  |  |  |  |  |  |  |  |
| 1 | Vitvi00g04651\_t001 |  | \| \| |  |  |  |  |  |  |  |  |  |  |
| 1 | Vitvi00g04652\_t001 |  | \| \| |  |  |  |  |  |  |  |  |  |  |
| 1 | Vitvi00g04653\_t001 |  | \| \| |  |  |  |  |  |  |  |  |  |  |
| 1 | Vitvi00g04654\_t002 |  | \| \| |  |  |  |  |  |  |  |  |  |  |
| 1 | Vitvi00g04655\_t001 |  | \| \| |  |  |  |  |  |  |  |  |  |  |
| 1 | Vitvi10g02309\_t001 |  | Vitvi10g04115\_t001 |  |  |  |  |  |  |  |  |  |  |
| 1 | Vitvi10g02308\_t001 |  | Vitvi10g01703\_t001 |  |  |  |  |  |  |  |  |  |  |
| 1 | Vitvi00g04656\_t001 |  | Vitvi10g00241\_t001 |  |  |  |  |  |  |  |  |  |  |
| 0 | Vitvi00g04657\_t001 |  |  |  |  |  |  |  |  |  |  |  |  |
| 0 | Vitvi00g04658\_t001 |  |  |  |  |  |  |  |  |  |  |  |  |
| 0 | Vitvi00g04659\_t001 |  |  |  |  |  |  |  |  |  |  |  |  |
| 0 | Vitvi00g00918\_t001 |  |  |  |  |  |  |  |  |  |  |  |  |
| 0 | Vitvi00g04660\_t001 |  |  |  |  |  |  |  |  |  |  |  |  |
| 0 | Vitvi00g04661\_t001 |  |  |  |  |  |  |  |  |  |  |  |  |
| 0 | Vitvi00g04662\_t001 |  |  |  |  |  |  |  |  |  |  |  |  |
| 0 | Vitvi00g04663\_t001 |  |  |  |  |  |  |  |  |  |  |  |  |
| 0 | Vitvi00g04664\_t001 |  |  |  |  |  |  |  |  |  |  |  |  |
| 0 | Vitvi00g04665\_t001 |  |  |  |  |  |  |  |  |  |  |  |  |
| 0 | Vitvi00g04666\_t001 |  |  |  |  |  |  |  |  |  |  |  |  |
| 0 | Vitvi00g04667\_t001 |  |  |  |  |  |  |  |  |  |  |  |  |
| 0 | Vitvi00g04668\_t001 |  |  |  |  |  |  |  |  |  |  |  |  |
| 0 | Vitvi00g04669\_t001 |  |  |  |  |  |  |  |  |  |  |  |  |
| 0 | Vitvi00g04670\_t001 |  |  |  |  |  |  |  |  |  |  |  |  |
| 0 | Vitvi00g04671\_t001 |  |  |  |  |  |  |  |  |  |  |  |  |
| 0 | Vitvi00g04672\_t001 |  |  |  |  |  |  |  |  |  |  |  |  |
| 0 | Vitvi00g04673\_t001 |  |  |  |  |  |  |  |  |  |  |  |  |
| 0 | Vitvi00g04674\_t001 |  |  |  |  |  |  |  |  |  |  |  |  |
| 0 | Vitvi00g04675\_t001 |  |  |  |  |  |  |  |  |  |  |  |  |
| 0 | Vitvi00g04676\_t001 |  |  |  |  |  |  |  |  |  |  |  |  |
| 0 | Vitvi00g04677\_t001 |  |  |  |  |  |  |  |  |  |  |  |  |
| 0 | Vitvi00g04678\_t001 |  |  |  |  |  |  |  |  |  |  |  |  |
| 0 | Vitvi00g04679\_t001 |  |  |  |  |  |  |  |  |  |  |  |  |
| 0 | Vitvi00g04680\_t001 |  |  |  |  |  |  |  |  |  |  |  |  |
| 0 | Vitvi00g04681\_t001 |  |  |  |  |  |  |  |  |  |  |  |  |
| 0 | Vitvi00g04682\_t001 |  |  |  |  |  |  |  |  |  |  |  |  |
| 0 | Vitvi00g04683\_t001 |  |  |  |  |  |  |  |  |  |  |  |  |
| 0 | Vitvi00g04684\_t001 |  |  |  |  |  |  |  |  |  |  |  |  |
| 0 | Vitvi00g04685\_t001 |  |  |  |  |  |  |  |  |  |  |  |  |
| 0 | Vitvi00g04686\_t001 |  |  |  |  |  |  |  |  |  |  |  |  |
| 0 | Vitvi00g04687\_t001 |  |  |  |  |  |  |  |  |  |  |  |  |
| 0 | Vitvi00g04688\_t001 |  |  |  |  |  |  |  |  |  |  |  |  |
| 0 | Vitvi00g04689\_t001 |  |  |  |  |  |  |  |  |  |  |  |  |
| 0 | Vitvi00g04690\_t001 |  |  |  |  |  |  |  |  |  |  |  |  |
| 0 | Vitvi00g04691\_t001 |  |  |  |  |  |  |  |  |  |  |  |  |
| 0 | Vitvi00g04692\_t001 |  |  |  |  |  |  |  |  |  |  |  |  |
| 0 | Vitvi00g04693\_t001 |  |  |  |  |  |  |  |  |  |  |  |  |
| 0 | Vitvi03g01878\_t001 |  |  |  |  |  |  |  |  |  |  |  |  |
| 0 | Vitvi03g01892\_t001 |  |  |  |  |  |  |  |  |  |  |  |  |
| 0 | Vitvi00g04694\_t001 |  |  |  |  |  |  |  |  |  |  |  |  |
| 0 | Vitvi00g04695\_t001 |  |  |  |  |  |  |  |  |  |  |  |  |
| 0 | Vitvi00g04696\_t001 |  |  |  |  |  |  |  |  |  |  |  |  |
| 0 | Vitvi00g04697\_t001 |  |  |  |  |  |  |  |  |  |  |  |  |
| 0 | Vitvi00g04698\_t001 |  |  |  |  |  |  |  |  |  |  |  |  |
| 0 | Vitvi00g04699\_t001 |  |  |  |  |  |  |  |  |  |  |  |  |
| 0 | Vitvi00g04700\_t001 |  |  |  |  |  |  |  |  |  |  |  |  |
| 0 | Vitvi00g04701\_t001 |  |  |  |  |  |  |  |  |  |  |  |  |
| 0 | Vitvi00g04702\_t001 |  |  |  |  |  |  |  |  |  |  |  |  |
| 0 | Vitvi00g04703\_t001 |  |  |  |  |  |  |  |  |  |  |  |  |
| 0 | Vitvi00g04704\_t001 |  |  |  |  |  |  |  |  |  |  |  |  |
| 0 | Vitvi00g04705\_t001 |  |  |  |  |  |  |  |  |  |  |  |  |
| 0 | Vitvi00g04706\_t001 |  |  |  |  |  |  |  |  |  |  |  |  |
| 0 | Vitvi00g04707\_t001 |  |  |  |  |  |  |  |  |  |  |  |  |
| 0 | Vitvi00g04708\_t001 |  |  |  |  |  |  |  |  |  |  |  |  |
| 0 | Vitvi00g04709\_t001 |  |  |  |  |  |  |  |  |  |  |  |  |
| 0 | Vitvi00g04710\_t001 |  |  |  |  |  |  |  |  |  |  |  |  |
| 0 | Vitvi00g04711\_t001 |  |  |  |  |  |  |  |  |  |  |  |  |
| 0 | Vitvi00g04712\_t001 |  |  |  |  |  |  |  |  |  |  |  |  |
| 0 | Vitvi00g04713\_t001 |  |  |  |  |  |  |  |  |  |  |  |  |
| 0 | Vitvi00g04714\_t001 |  |  |  |  |  |  |  |  |  |  |  |  |
| 0 | Vitvi00g04715\_t001 |  |  |  |  |  |  |  |  |  |  |  |  |
| 0 | Vitvi00g04716\_t001 |  |  |  |  |  |  |  |  |  |  |  |  |
| 0 | Vitvi00g04717\_t001 |  |  |  |  |  |  |  |  |  |  |  |  |
| 0 | Vitvi00g04718\_t001 |  |  |  |  |  |  |  |  |  |  |  |  |
| 0 | Vitvi00g04719\_t001 |  |  |  |  |  |  |  |  |  |  |  |  |
| 0 | Vitvi00g04720\_t001 |  |  |  |  |  |  |  |  |  |  |  |  |
| 0 | Vitvi00g04721\_t001 |  |  |  |  |  |  |  |  |  |  |  |  |
| 0 | Vitvi00g04722\_t001 |  |  |  |  |  |  |  |  |  |  |  |  |
| 0 | Vitvi00g04723\_t001 |  |  |  |  |  |  |  |  |  |  |  |  |
| 0 | Vitvi00g04724\_t001 |  |  |  |  |  |  |  |  |  |  |  |  |
| 0 | Vitvi00g04725\_t001 |  |  |  |  |  |  |  |  |  |  |  |  |
| 0 | Vitvi00g04726\_t001 |  |  |  |  |  |  |  |  |  |  |  |  |
| 0 | Vitvi00g04727\_t001 |  |  |  |  |  |  |  |  |  |  |  |  |
| 0 | Vitvi00g04728\_t001 |  |  |  |  |  |  |  |  |  |  |  |  |
| 0 | Vitvi00g04729\_t001 |  |  |  |  |  |  |  |  |  |  |  |  |
| 0 | Vitvi00g04730\_t001 |  |  |  |  |  |  |  |  |  |  |  |  |
| 0 | Vitvi00g04731\_t001 |  |  |  |  |  |  |  |  |  |  |  |  |
| 0 | Vitvi00g04732\_t001 |  |  |  |  |  |  |  |  |  |  |  |  |
| 0 | Vitvi00g04733\_t001 |  |  |  |  |  |  |  |  |  |  |  |  |
| 0 | Vitvi00g04734\_t001 |  |  |  |  |  |  |  |  |  |  |  |  |
| 0 | Vitvi00g04735\_t001 |  |  |  |  |  |  |  |  |  |  |  |  |
| 0 | Vitvi00g04736\_t001 |  |  |  |  |  |  |  |  |  |  |  |  |
| 0 | Vitvi00g04737\_t001 |  |  |  |  |  |  |  |  |  |  |  |  |
| 0 | Vitvi00g04738\_t001 |  |  |  |  |  |  |  |  |  |  |  |  |
| 0 | Vitvi00g04739\_t001 |  |  |  |  |  |  |  |  |  |  |  |  |
| 0 | Vitvi00g04740\_t001 |  |  |  |  |  |  |  |  |  |  |  |  |
| 0 | Vitvi00g04741\_t001 |  |  |  |  |  |  |  |  |  |  |  |  |
| 0 | Vitvi00g04742\_t001 |  |  |  |  |  |  |  |  |  |  |  |  |
| 0 | Vitvi00g04743\_t001 |  |  |  |  |  |  |  |  |  |  |  |  |
| 0 | Vitvi00g04744\_t001 |  |  |  |  |  |  |  |  |  |  |  |  |
| 0 | Vitvi00g04745\_t001 |  |  |  |  |  |  |  |  |  |  |  |  |
| 0 | Vitvi00g04746\_t001 |  |  |  |  |  |  |  |  |  |  |  |  |
| 0 | Vitvi00g04747\_t001 |  |  |  |  |  |  |  |  |  |  |  |  |
| 0 | Vitvi00g04748\_t001 |  |  |  |  |  |  |  |  |  |  |  |  |
| 0 | Vitvi00g04749\_t001 |  |  |  |  |  |  |  |  |  |  |  |  |
| 0 | Vitvi00g04750\_t001 |  |  |  |  |  |  |  |  |  |  |  |  |
| 0 | Vitvi00g04751\_t001 |  |  |  |  |  |  |  |  |  |  |  |  |
| 0 | Vitvi00g04752\_t001 |  |  |  |  |  |  |  |  |  |  |  |  |
| 0 | Vitvi00g04753\_t001 |  |  |  |  |  |  |  |  |  |  |  |  |
| 0 | Vitvi00g04754\_t001 |  |  |  |  |  |  |  |  |  |  |  |  |
| 0 | Vitvi00g04755\_t001 |  |  |  |  |  |  |  |  |  |  |  |  |
| 0 | Vitvi00g04756\_t001 |  |  |  |  |  |  |  |  |  |  |  |  |
| 0 | Vitvi00g04757\_t001 |  |  |  |  |  |  |  |  |  |  |  |  |
| 0 | Vitvi00g04758\_t001 |  |  |  |  |  |  |  |  |  |  |  |  |
| 0 | Vitvi00g04759\_t001 |  |  |  |  |  |  |  |  |  |  |  |  |
| 0 | Vitvi00g04760\_t001 |  |  |  |  |  |  |  |  |  |  |  |  |
| 0 | Vitvi00g04761\_t001 |  |  |  |  |  |  |  |  |  |  |  |  |
| 0 | Vitvi00g04762\_t001 |  |  |  |  |  |  |  |  |  |  |  |  |
| 0 | Vitvi00g04763\_t001 |  |  |  |  |  |  |  |  |  |  |  |  |
| 0 | Vitvi00g04764\_t001 |  |  |  |  |  |  |  |  |  |  |  |  |
| 0 | Vitvi00g04765\_t001 |  |  |  |  |  |  |  |  |  |  |  |  |
| 0 | Vitvi00g04766\_t001 |  |  |  |  |  |  |  |  |  |  |  |  |
| 0 | Vitvi00g04767\_t001 |  |  |  |  |  |  |  |  |  |  |  |  |
| 0 | Vitvi00g04768\_t001 |  |  |  |  |  |  |  |  |  |  |  |  |
| 0 | Vitvi00g04769\_t001 |  |  |  |  |  |  |  |  |  |  |  |  |
| 0 | Vitvi07g03113\_t001 |  |  |  |  |  |  |  |  |  |  |  |  |
| 0 | Vitvi00g04770\_t001 |  |  |  |  |  |  |  |  |  |  |  |  |
| 0 | Vitvi00g04771\_t001 |  |  |  |  |  |  |  |  |  |  |  |  |
| 0 | Vitvi00g04772\_t001 |  |  |  |  |  |  |  |  |  |  |  |  |
| 0 | Vitvi00g04773\_t001 |  |  |  |  |  |  |  |  |  |  |  |  |
| 0 | Vitvi10g02364\_t001 |  |  |  |  |  |  |  |  |  |  |  |  |
| 0 | Vitvi00g04774\_t001 |  |  |  |  |  |  |  |  |  |  |  |  |
| 0 | Vitvi00g04775\_t001 |  |  |  |  |  |  |  |  |  |  |  |  |
| 0 | Vitvi07g03115\_t001 |  |  |  |  |  |  |  |  |  |  |  |  |
| 0 | Vitvi00g04776\_t001 |  |  |  |  |  |  |  |  |  |  |  |  |
| 0 | Vitvi00g04777\_t001 |  |  |  |  |  |  |  |  |  |  |  |  |
| 0 | Vitvi00g04778\_t001 |  |  |  |  |  |  |  |  |  |  |  |  |
| 0 | Vitvi00g04779\_t001 |  |  |  |  |  |  |  |  |  |  |  |  |
| 0 | Vitvi00g04780\_t001 |  |  |  |  |  |  |  |  |  |  |  |  |
| 0 | Vitvi00g04781\_t001 |  |  |  |  |  |  |  |  |  |  |  |  |
| 0 | Vitvi00g04782\_t001 |  |  |  |  |  |  |  |  |  |  |  |  |
| 0 | Vitvi00g04783\_t001 |  |  |  |  |  |  |  |  |  |  |  |  |
| 0 | Vitvi00g04784\_t001 |  |  |  |  |  |  |  |  |  |  |  |  |
| 0 | Vitvi00g04785\_t001 |  |  |  |  |  |  |  |  |  |  |  |  |
| 0 | Vitvi15g01768\_t001 |  |  |  |  |  |  |  |  |  |  |  |  |
| 0 | Vitvi00g04786\_t001 |  |  |  |  |  |  |  |  |  |  |  |  |
| 0 | Vitvi00g04787\_t001 |  |  |  |  |  |  |  |  |  |  |  |  |
| 0 | Vitvi07g03119\_t001 |  |  |  |  |  |  |  |  |  |  |  |  |
| 0 | Vitvi00g04788\_t001 |  |  |  |  |  |  |  |  |  |  |  |  |
| 0 | Vitvi00g04789\_t001 |  |  |  |  |  |  |  |  |  |  |  |  |
| 0 | Vitvi00g04790\_t001 |  |  |  |  |  |  |  |  |  |  |  |  |
| 0 | Vitvi00g04791\_t001 |  |  |  |  |  |  |  |  |  |  |  |  |
| 0 | Vitvi13g02599\_t001 |  |  |  |  |  |  |  |  |  |  |  |  |
| 0 | Vitvi00g04792\_t001 |  |  |  |  |  |  |  |  |  |  |  |  |
| 0 | Vitvi05g02286\_t001 |  |  |  |  |  |  |  |  |  |  |  |  |
| 0 | Vitvi10g02384\_t001 |  |  |  |  |  |  |  |  |  |  |  |  |
| 0 | Vitvi00g04793\_t001 |  |  |  |  |  |  |  |  |  |  |  |  |
| 0 | Vitvi16g02146\_t001 |  |  |  |  |  |  |  |  |  |  |  |  |
| 0 | Vitvi00g04794\_t001 |  |  |  |  |  |  |  |  |  |  |  |  |
| 0 | Vitvi00g04795\_t001 |  |  |  |  |  |  |  |  |  |  |  |  |
| 0 | Vitvi00g04796\_t001 |  |  |  |  |  |  |  |  |  |  |  |  |
| 0 | Vitvi00g04797\_t001 |  |  |  |  |  |  |  |  |  |  |  |  |
| 0 | Vitvi00g04798\_t001 |  |  |  |  |  |  |  |  |  |  |  |  |
| 0 | Vitvi00g04799\_t001 |  |  |  |  |  |  |  |  |  |  |  |  |
| 0 | Vitvi00g04800\_t001 |  |  |  |  |  |  |  |  |  |  |  |  |
| 0 | Vitvi00g04801\_t001 |  |  |  |  |  |  |  |  |  |  |  |  |
| 0 | Vitvi00g04802\_t001 |  |  |  |  |  |  |  |  |  |  |  |  |
| 0 | Vitvi00g04803\_t001 |  |  |  |  |  |  |  |  |  |  |  |  |
| 0 | Vitvi00g04804\_t001 |  |  |  |  |  |  |  |  |  |  |  |  |
| 0 | Vitvi00g04805\_t001 |  |  |  |  |  |  |  |  |  |  |  |  |
| 0 | Vitvi00g04806\_t001 |  |  |  |  |  |  |  |  |  |  |  |  |
| 0 | Vitvi00g01122\_t001 |  |  |  |  |  |  |  |  |  |  |  |  |
| 0 | Vitvi00g02171\_t001 |  |  |  |  |  |  |  |  |  |  |  |  |
| 0 | Vitvi00g02172\_t001 |  |  |  |  |  |  |  |  |  |  |  |  |
| 0 | Vitvi00g04807\_t001 |  |  |  |  |  |  |  |  |  |  |  |  |
| 0 | Vitvi00g04808\_t001 |  |  |  |  |  |  |  |  |  |  |  |  |
| 0 | Vitvi00g04809\_t001 |  |  |  |  |  |  |  |  |  |  |  |  |
| 0 | Vitvi00g04810\_t001 |  |  |  |  |  |  |  |  |  |  |  |  |
| 0 | Vitvi00g04811\_t001 |  |  |  |  |  |  |  |  |  |  |  |  |
| 0 | Vitvi00g04812\_t001 |  |  |  |  |  |  |  |  |  |  |  |  |
| 0 | Vitvi00g04813\_t001 |  |  |  |  |  |  |  |  |  |  |  |  |
| 0 | Vitvi00g04814\_t001 |  |  |  |  |  |  |  |  |  |  |  |  |
| 0 | Vitvi00g04815\_t001 |  |  |  |  |  |  |  |  |  |  |  |  |
| 0 | Vitvi00g04816\_t001 |  |  |  |  |  |  |  |  |  |  |  |  |
| 0 | Vitvi00g04817\_t001 |  |  |  |  |  |  |  |  |  |  |  |  |
| 0 | Vitvi00g04818\_t001 |  |  |  |  |  |  |  |  |  |  |  |  |
| 0 | Vitvi00g04819\_t001 |  |  |  |  |  |  |  |  |  |  |  |  |
| 0 | Vitvi00g04820\_t001 |  |  |  |  |  |  |  |  |  |  |  |  |
| 0 | Vitvi00g04821\_t001 |  |  |  |  |  |  |  |  |  |  |  |  |
| 0 | Vitvi00g04822\_t001 |  |  |  |  |  |  |  |  |  |  |  |  |
| 0 | Vitvi00g04823\_t001 |  |  |  |  |  |  |  |  |  |  |  |  |
| 0 | Vitvi00g04824\_t001 |  |  |  |  |  |  |  |  |  |  |  |  |
| 0 | Vitvi00g04825\_t001 |  |  |  |  |  |  |  |  |  |  |  |  |
| 0 | Vitvi00g04826\_t001 |  |  |  |  |  |  |  |  |  |  |  |  |
| 0 | Vitvi00g04827\_t001 |  |  |  |  |  |  |  |  |  |  |  |  |
| 0 | Vitvi00g04828\_t001 |  |  |  |  |  |  |  |  |  |  |  |  |
| 0 | Vitvi00g04829\_t001 |  |  |  |  |  |  |  |  |  |  |  |  |
| 0 | Vitvi00g04830\_t001 |  |  |  |  |  |  |  |  |  |  |  |  |
| 0 | Vitvi00g04831\_t001 |  |  |  |  |  |  |  |  |  |  |  |  |
| 0 | Vitvi00g04832\_t001 |  |  |  |  |  |  |  |  |  |  |  |  |
| 0 | Vitvi00g04833\_t001 |  |  |  |  |  |  |  |  |  |  |  |  |
| 0 | Vitvi00g04834\_t001 |  |  |  |  |  |  |  |  |  |  |  |  |
| 0 | Vitvi00g04835\_t001 |  |  |  |  |  |  |  |  |  |  |  |  |
| 0 | Vitvi00g04836\_t001 |  |  |  |  |  |  |  |  |  |  |  |  |
| 0 | Vitvi00g04837\_t001 |  |  |  |  |  |  |  |  |  |  |  |  |
| 0 | Vitvi00g04838\_t001 |  |  |  |  |  |  |  |  |  |  |  |  |
| 0 | Vitvi00g04839\_t002 |  |  |  |  |  |  |  |  |  |  |  |  |
| 0 | Vitvi00g04840\_t001 |  |  |  |  |  |  |  |  |  |  |  |  |
| 0 | Vitvi00g04841\_t001 |  |  |  |  |  |  |  |  |  |  |  |  |
| 0 | Vitvi00g04842\_t001 |  |  |  |  |  |  |  |  |  |  |  |  |
| 0 | Vitvi00g04843\_t001 |  |  |  |  |  |  |  |  |  |  |  |  |
| 0 | Vitvi00g04844\_t001 |  |  |  |  |  |  |  |  |  |  |  |  |
| 0 | Vitvi00g04845\_t001 |  |  |  |  |  |  |  |  |  |  |  |  |
| 0 | Vitvi00g04846\_t001 |  |  |  |  |  |  |  |  |  |  |  |  |
| 0 | Vitvi00g04847\_t001 |  |  |  |  |  |  |  |  |  |  |  |  |
| 0 | Vitvi00g04848\_t001 |  |  |  |  |  |  |  |  |  |  |  |  |
| 0 | Vitvi00g04849\_t001 |  |  |  |  |  |  |  |  |  |  |  |  |
| 0 | Vitvi00g04850\_t001 |  |  |  |  |  |  |  |  |  |  |  |  |
| 0 | Vitvi00g04851\_t001 |  |  |  |  |  |  |  |  |  |  |  |  |
| 0 | Vitvi00g04852\_t001 |  |  |  |  |  |  |  |  |  |  |  |  |
| 0 | Vitvi00g04853\_t001 |  |  |  |  |  |  |  |  |  |  |  |  |
| 0 | Vitvi00g04854\_t001 |  |  |  |  |  |  |  |  |  |  |  |  |
| 0 | Vitvi00g04855\_t001 |  |  |  |  |  |  |  |  |  |  |  |  |
| 0 | Vitvi00g04856\_t001 |  |  |  |  |  |  |  |  |  |  |  |  |
| 0 | Vitvi00g04857\_t001 |  |  |  |  |  |  |  |  |  |  |  |  |
| 0 | Vitvi00g04858\_t001 |  |  |  |  |  |  |  |  |  |  |  |  |
| 0 | Vitvi00g04859\_t001 |  |  |  |  |  |  |  |  |  |  |  |  |
| 0 | Vitvi00g04860\_t001 |  |  |  |  |  |  |  |  |  |  |  |  |
| 0 | Vitvi00g04861\_t001 |  |  |  |  |  |  |  |  |  |  |  |  |
| 0 | Vitvi00g04862\_t001 |  |  |  |  |  |  |  |  |  |  |  |  |
| 0 | Vitvi00g04863\_t001 |  |  |  |  |  |  |  |  |  |  |  |  |
| 0 | Vitvi00g04864\_t001 |  |  |  |  |  |  |  |  |  |  |  |  |
| 0 | Vitvi00g04865\_t001 |  |  |  |  |  |  |  |  |  |  |  |  |
| 0 | Vitvi00g04866\_t001 |  |  |  |  |  |  |  |  |  |  |  |  |
| 0 | Vitvi00g04867\_t001 |  |  |  |  |  |  |  |  |  |  |  |  |
| 0 | Vitvi00g04868\_t001 |  |  |  |  |  |  |  |  |  |  |  |  |
| 0 | Vitvi00g04869\_t001 |  |  |  |  |  |  |  |  |  |  |  |  |
| 0 | Vitvi00g04870\_t001 |  |  |  |  |  |  |  |  |  |  |  |  |
| 0 | Vitvi00g04871\_t001 |  |  |  |  |  |  |  |  |  |  |  |  |
| 0 | Vitvi00g04872\_t001 |  |  |  |  |  |  |  |  |  |  |  |  |
| 0 | Vitvi00g04873\_t001 |  |  |  |  |  |  |  |  |  |  |  |  |
| 0 | Vitvi00g04874\_t001 |  |  |  |  |  |  |  |  |  |  |  |  |
| 0 | Vitvi00g04875\_t001 |  |  |  |  |  |  |  |  |  |  |  |  |
| 0 | Vitvi00g04876\_t001 |  |  |  |  |  |  |  |  |  |  |  |  |
| 0 | Vitvi00g04877\_t001 |  |  |  |  |  |  |  |  |  |  |  |  |
| 0 | Vitvi00g04878\_t001 |  |  |  |  |  |  |  |  |  |  |  |  |
| 0 | Vitvi00g04879\_t001 |  |  |  |  |  |  |  |  |  |  |  |  |
| 0 | Vitvi00g04880\_t001 |  |  |  |  |  |  |  |  |  |  |  |  |
| 0 | Vitvi00g04881\_t001 |  |  |  |  |  |  |  |  |  |  |  |  |
| 0 | Vitvi00g04882\_t001 |  |  |  |  |  |  |  |  |  |  |  |  |
| 0 | Vitvi00g04883\_t001 |  |  |  |  |  |  |  |  |  |  |  |  |
